# Transformation to small cell lung cancer is irrespective of *EGFR* and accelerated by SMAD4-mediated *ASCL1* transcription independently of *RB1* in non-small cell lung cancer

**DOI:** 10.1186/s12964-023-01260-8

**Published:** 2024-01-17

**Authors:** Xi Ding, Min-xing Shi, Di Liu, Jing-xue Cao, Kai-xuan Zhang, Run-dong Zhang, Li-ping Zhang, Kai-xing Ai, Bo Su, Jie Zhang

**Affiliations:** 1 Department of Central Laboratory, Shanghai Pulmonary Hospital, School of Medicine, Tongji University, Shanghai, 200092 China; 2 Department of Respiratory Medicine, Shanghai Pulmonary Hospital, School of Medicine, Tongji University, Shanghai, 200092 China; 3 Department of Radiotherapy, Shanghai Pulmonary Hospital, School of Medicine, Tongji University, Shanghai, 200092 China; 4 Department of Radiology, Shanghai Pulmonary Hospital, School of Medicine, Tongji University, Shanghai, 200092 China; 5 Department of Thoracic Surgery, Shanghai Pulmonary Hospital, School of Medicine, Tongji University, Shanghai, 200092 China; 6 Department of General Surgery, Shanghai Pulmonary Hospital, School of Medicine, Tongji University, Shanghai, 200092 China; 7 Department of Pathology, Shanghai Pulmonary Hospital, School of Medicine, Tongji University, Shanghai, 200092 China; 8 Department of Oncology, Shanghai Pulmonary Hospital, School of Medicine, Tongji University, Shanghai, 200092 China

**Keywords:** SMAD4, Small-cell transformation, EGFR-TKI therapy, Drug resistance, Myc inhibitor

## Abstract

**Objectives:**

Histological transformation to small cell lung cancer (SCLC) has been identified as a mechanism of TKIs resistance in *EGFR*-mutant non-small cell lung cancer (NSCLC). We aim to explore the prevalence of transformation in *EGFR*-wildtype NSCLC and the mechanism of SCLC transformation, which are rarely understood.

**Methods:**

We reviewed 1474 NSCLC patients to investigate the NSCLC-to-SCLC transformed cases and the basic clinical characteristics, driver gene status and disease course of them. To explore the potential functional genes in SCLC transformation, we obtained pre- and post-transformation specimens and subjected them to a multigene NGS panel involving 416 cancer-related genes. To validate the putative gene function, we established knocked-out models by CRISPR-Cas 9 in HCC827 and A549-TP53^-/-^ cells and investigated the effects on tumor growth, drug sensitivity and neuroendocrine phenotype in vitro and in vivo. We also detected the expression level of protein and mRNA to explore the molecular mechanism involved.

**Results:**

We firstly reported an incidence rate of 9.73% (11/113) of SCLC transformation in *EGFR*-wildtype NSCLC and demonstrated that SCLC transformation is irrespective of *EGFR* mutation status (*P* = 0.16). We sequenced 8 paired tumors and identified a series of mutant genes specially in transformed SCLC such as *SMAD4*, *RICTOR* and *RET*. We firstly demonstrated that *SMAD4* deficiency can accelerate SCLC transition by inducing neuroendocrine phenotype regardless of *RB1* status in *TP53*-deficient NSCLC cells. Further mechanical experiments identified the SMAD4 can regulate *ASCL1* transcription competitively with Myc in NSCLC cells and Myc inhibitor acts as a potential subsequent treatment agent.

**Conclusions:**

Transformation to SCLC is irrespective of *EFGR* status and can be accelerated by SMAD4 in non-small cell lung cancer. Myc inhibitor acts as a potential therapeutic drug for SMAD4-mediated resistant lung cancer.

Video Abstract

**Supplementary Information:**

The online version contains supplementary material available at 10.1186/s12964-023-01260-8.

## Introduction

Histological transformation to small cell lung cancer (SCLC), particularly from *EGFR* mutant lung adenocarcinoma (LUAD), has been recognized as a mechanism of EGFR tyrosine kinase inhibitors (EGFR TKIs) resistance as listed in the NCCN (National Comprehensive Cancer Network) clinical practice guidelines for non-small cell lung cancer (NSCLC) (https://www.nccn.org/, 2021.V3) [1, 2]. However, there still remain several critical questions about this phenomenon. One major topic is, case reports suggested that SCLC transformation can also occur after ALK inhibitor treatment [3, 4],PD1/PD-L1 immunotherapy [5], which implies that SCLC transformation is not restricted in *EGFR* mutant LUAD, while systematic real-world survey with a larger cohort is lacking.

Another issue that we are concerned about is the mechanism and the subsequent strategies of SCLC transition. *RB1* and *TP53* abnormalities are essentially universal in de-novo SCLCs [6] and also seem to be critical in transformed SCLC. Lee et al. [7]demonstrated that early alteration of *RB1* and *TP53* in primary LUAD indicated a high tendency of SCLC transition. Matthew et al. [8] verified that *RB1* deletion is required for NSCLC-to-SCLC conversion because almost all of the transformed SCLCs exhibited homozygous deletion of the *RB1* gene. Meanwhile, in Matthew’s work, *RB1* deletion was also observed in LUAD where TKI resistance occurred independently of SCLC transformation, suggesting that *RB1* deletion alone is insufficient to explain the phenotypic switch [8]. Besides *TP53* and *RB1*, there’s still lack of understanding of involved genes in SCLC transformation.

As reported, 3–14% [2, 9] of EGFR-TKIs treated LUAD patients develop SCLC transformation and once transformation occurs, the treatment strategy is extremely limited coming with poor prognosis. Léonie Ferrer et al. [10] reported a 10-month median overall survival (mOS) in 61 transformed cases. Nicolas Marcoux et al. [11] reported a 10.9-month mOS with frequent CNS metastases in 57 SCLC transformed cases. Almost all transformed cases were treated with platinum and etoposide (EP), which is the classic therapy of SCLC. Marcoux’s study also reported that transformed SCLC showed unsatisfactory response to PD-1 antibodies. Thus, figuring out the mechanism and the involved key regulators of SCLC transformation is urgently demanded for new therapeutic drugs development.

Hence, in this real-world study, we investigated the occurrence of SCLC transformation in a relatively large NSCLC cohort. We also performed next-generation sequencing (NGS) on the paired tumor samples obtained from transformed cases to explore the potential genes contributing to the histological transformation.

## Materials and methods

### Patients’ clinicopathological characteristics

All participating NSCLC patients were pathologically diagnosed at Shanghai Pulmonary Hospital with key driver genes detection including *EGFR, KRAS*, and *BRAF* mutation and *ALK*, and *ROS1* fusion by ARMS-PCR (AmoyDx, Xiamen, China) between January 2013 and December 2016. For all cases, clinical data (gender, age, clinical stage, smoking history and ECOG PS) were collected at the entry time. The pathological histology was determined with available surgical resected sample, biopsy or re-biopsy sample during the whole follow-up. EGFR-TKI therapies in this study included gefitinib and erlotinib. The short-term responses were evaluated after the first 2 cycles for chemotherapy, and after the first month for targeted therapy according to the Response Evaluation Criteria in Solid Tumors (RECIST). Progression-free survival (PFS) was defined as the period from the date of receiving treatment to that of objective disease progression or death or the last follow-up, and OS was defined as the period from the date of receiving treatment to that of death or the last follow-up.

### Targeted tumor gene panel test using next generation sequencing

The paired tumor formalin-fixed paraffin-embedding (FFPE) specimens from resected tumor or biopsy were subjected to a tumor multigene NGS panel covering the whole exons and significant introns of 416 cancer-related genes (Nanjing Geneseeq Technology Inc., Jiangsu, China, CAP/CLIA-certified). Genomic DNA was extracted (QIAamp DNA FFPE Tissue Kit, QIAGEN) and quality controlled. The sequencing libraries were prepared with the optimized protocols according to the manufacturer’s instructions (GeneseeqPrime, Jiangsu, China). The capture-enriched libraries were sequenced on Illumina HiSeq 4000 platform. Single nucleotide variation (SNVs) and indels were called by VarScan2 and Haplotype Caller/Unified Genotyper in GATK and filtered out by dbSNP database and data from 1000 Genome project. Gene fusions were identified by FACTERA and copy number variations (CNVs) were measured with ADTEx.

### Cell culture

The human NSCLC cell line HCC827 was purchased from ATCC, and A549-TP53-/- was previously developed and preserved in the laboratory. Cells were cultured in DMEM (HyClone Lot. SH30585.02) supplemented with 10% FBS (Gibco Lot. 10,100,147) and 100U/ml penicillin/streptomycin (Gibco Lot. 15,140,122) at 37 °C in 5% CO2 atmosphere.

### Establishment of *SMAD4* and *RB1* knocking-out cell lines

*SMAD4* and *RB1* genes were knocked out by CRISPR-Cas9 technique (Genomeditech Shanghai, Co., LTD). SgRNA primers were designed on Zhang Feng’s laboratory website: *SMAD4*, AGAGCAGGACAGCGGCCCGG and *RB1*, AGAGCAGGACAGCGGCCCGG. Monocolony screening was conducted to select the homozygotes-deleted clones after stable transfection lines were established. The cells were counted and diluted to 1 cell per 100ul of cell suspension and cultured at 100ul volume per well in 96-well cell plates. After regular incubation for 2 days, the cell clusters were observed under microscope and the single clones were transferred to 6 or 10 cm petri dish for further culture and proteins were extracted when cell amounts were huge.

### MTT assay

For cell growth ability, cells were plated into 96-well plates at a density of 1000 cells per well. Culture medium were stripped off at different time points (24, 48,72, 96, 120, and 144 h) and cells were treated with 20 µl of MTT solution (5 mg/ml) at 37 ˚C for 4 h. Then 200 µl of DMSO was added to each well. The optical density (OD) was measured at 570-nm wavelength and each experiment was performed in triplicate. For drug sensitivity, cells were plated into 96-well plates at a density of 5000 cells per well and treated with drugs at different dilutions. Cells were cultured for 72 h and then culture medium were stripped off. MTT solution and DMSO and also OD measurement were then processed as described above.

### Luciferase reporter gene assay and ChIP assay

The luciferase reporter plasmid under the control of ASCL1 promoter was constructed by Genomeditech, Shanghai, China. Briefly, the − 2000 to − 1 upstream area of the human ASCL1 gene was amplified by PCR using human genomic DNA and a set of forward and reverse primers containing custom MluI and XhoI restriction enzyme sites, respectively. The PCR product was cloned into to pGL3-Basic, which is a promoter-less luciferase vector. The PCR product was named as pGL3-Basic-H_ASCL1 promoter and the sequence was confirmed by sequencing analyses. The cells were transfected with pGL3-Basic-H_ASCL1 promoter plasmids, and after a transfection period of 24 h, the cells and lysates were collected. A Dual-Luciferase Reporter Assay System (Promega) was used to quantify luciferase activities following the manufacturer’s instructions. Firefly luciferase activity was normalized to Renilla luciferase activity. ChIP was performed following protocol of the ChIP assay kit purchased from Beyotime (Lot. P2078), and the antibodies used were as follows: SMAD4 (D3R4N) (CST #46,535) and MYC (Y69) (Abcam #ab32072). The PCR primer of *ASCL1* was forward-AGCCATTTGTCCCTCCTGTG, reverse-CTCCTCTTACCTCTTCCTCCC.

### Nude mouse tumor xenograft model

The BALB/C nude mice (Female, 6-week-old) were randomly divided into four groups (n = 4 per group) and cells (4 × 106) were subcutaneously injected into the unilateral hind limbs of the nude mice. Tumor size was measured for 6 weeks after inoculation to calculating tumor volume using the equation (length × width2/2). Animals were killed 6 weeks after inoculation, and the tumors were then excised for further usage.

### H&E staining, immunohistochemistry staining and Western blotting

Each resected tumor, biopsy or re-biopsy specimen from any primary tumor site, lymph node or metastases was examined with Hematoxylin-eosin (H&E) and immunohistochemistry (IHC) staining following regular protocols for histopathological observation. The histology for each specimen was determined independently by at least two pathology experts in the hospital. Cells were lysed with RIPA buffer containing a proteinase inhibitor mixture (Beyotime, P1005) and proteins were qualified by BCA assay (Beyotime, P0012s). Then western blot assay was performed following routine protocol. Antibodies used were listed in supplementary file.

### Statistical analysis

All statistical analyses were conducted on SPSS platform (SPSS Inc., Chicago, IL, version 20.0), and the graphs were achieved on Graphpad (Prisim 9) platform. The differences of continuous variables were evaluated by ANOVA’s test or t test. The relationships between classified variables were analyzed using χ2 test. A *P* value < 0.05 at both sides was considered as statistically significant. The motif analysis was performed on ConTra v3 platform. The relevant parameters were set as follows: Reference organism: Homo sapiens, Genomic position: chr12:102955674–102,957,673, and Stringency: core = 0.95, similarity matrix = 0.85.

## Results

### SCLC transformation is irrespective of *EGFR* mutation status or histological subtype in NSCLC with poor prognosis and acquired neuroendocrine phenotype

**Fig. 1:**
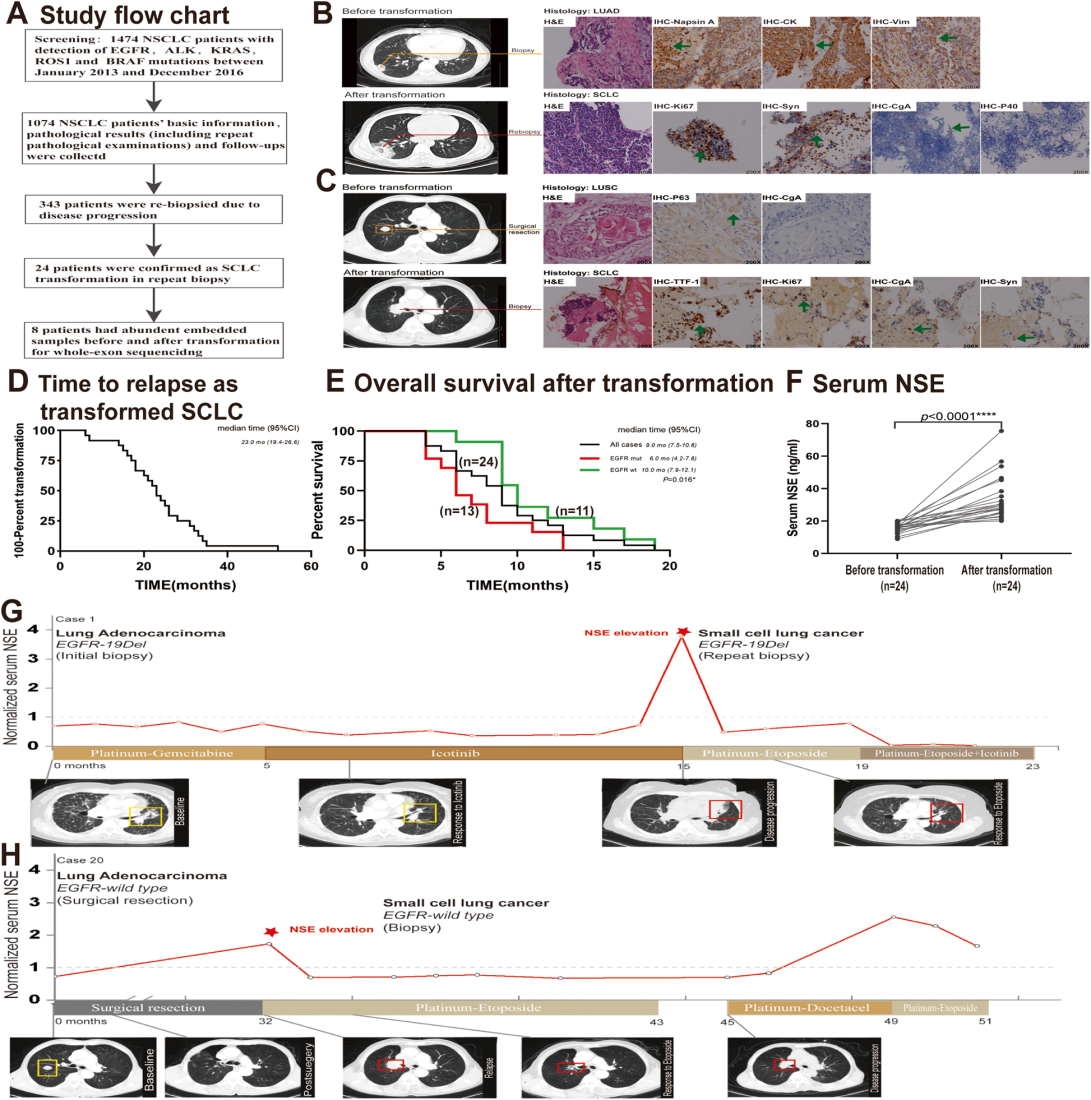
Study design and overall review of NSCLC-to-SCLC transformation in this study. **A** Study flow chart of this study. A total of 1474 NSCLC was reviewed, and 1074 patients were finally analyzed with further treatment in our institution were excluded. 24 cases were confirmed as SCLC transformation out of 343 patients with repeat biopsy, and 8 transformed cases with abundant tumor samples were further sequenced. **B** Typical images of SCLC transformation cases from lung adenocarcinoma (LUAD). This case presented with morphology of LUAD with positive IHC markers of Napsin A, CK and Vimentin at primary diagnosis before transformation. CT scan indicated tumor progression and the progressed tumor lesion showed morphology of SCLC with high index of Ki-67 and positive expression of Syn, CgA and negative expression of P40 at repeat biopsy (after transformation). **C** Typical images of SCLC transformation cases from lung squamous carcinoma (LUSC). This case underwent surgical resection and postoperative pathology supported the diagnosis of LUSC with morphological features and positive expression of P63 and negative expression of neuroendocrine marker, CgA at primary diagnosis before transformation. A relapsed tumor was observed on CT scan and the replased tumor showed morphology of SCLC with high index of Ki-67 and positive expression of TTF-1, CgA and Syn at repeat biopsy (after transformation). **D** Median transformation time in our study was 23.0 months (95%CI, 19.4–26.6). **E** Overall survival curve of 24 cases after transformation. After transformation developed, the mOS was 9.0 months (95%CI, 7.5–10.6), and the OS of EGFR wild type group was 4.0 months longer than that of EGFR mutant transformation cases (10.0 vs. 6.0 months, *P* = 0.016). **F-H** NSE elevation was observed in all transformation cases and representative images of NSE changes during whole disease course. **F** Comparison of serum NSE before and after SCLC transformation in 24 cases; **G-H** Dynamic serum NSE change in disease course of representative SCLC transformation cases

One thousand four hundred seventy-four NSCLC patients were firstly diagnosed and 1074 with further treatment in our institution were further analyzed (Fig. 1A; Table 1). To our concern, 343 NSCLC patients received re-biopsy and 24 SCLC transformation cases were confirmed (24/343, 7.0%) including 13 *EGFR* mutant, 2 with positive *ALK* rearrangement and 9 wildtypes for either detected driver genes (Table 2). In our study, the incidence rate of SCLC transformation was 5.65% (13/230) in *EGFR* mutant cohort and 9.73% (11/113) in *EGFR* wildtype cohort(*P* = 0.16). The prevalence of SCLC transformation was 7.5% (21/279) in LUAD, and 4.7% (3/64) in LUSC, respectively (*P* = 0.41). Our results revealed that SCLC transformation occurs regardless of *EGFR* mutation status or primary histological subtype (*P* > 0.05, Table 1). Two representative transformation cases were presented in Fig. 1B and C.

**Table 1:** General information of studies cases and the associations between clinical variables, SCLC transformation and EGFR mutation status in non-small cell lung cancer

		All patients (%)	*EGFR status*			
Variables			Mutant (*N* = 484)	Wild type (*N* = 590)	χ2	*P* value
**Gender**	**Male**	617(57.4%)	246(39.9%)	371(60.1%)	15.25	< 0.0001****
	**Female**	457(42.6%)	237(51.9%)	220(48.1%)		
**Age**	**≤ 60**	417(38.8%)	199(47.6%)	219(52.4%)	1.92	0.17
	**> 60**	657(61.2%)	284(43.3%)	372(56.7%)		
**Clinical stage**	**I-II**	599(55.8%)	268(44.7%)	331(55.3%)	4.37	0.22
	**IIIA**	159(14.8%)	62(39.0%)	97(61.0%)		
	**IIIB**	95(8.8%)	43(45.3%)	52(54.7%)		
	**IV**	221(20.6%)	110(49.8%)	111(50.2%)		
**Operation history**	**Yes**	738(68.7%)	330(44.7%)	408(55.3%)	0.06	0.80
	**No**	336(31.3%)	153(45.5%)	183(54.5%)		
**Histological type**	**Adenocarcinoma**	868(80.8%)	467(53.8%)	401(46.2%)	142.57	< 0.0001****
	**Squamous carcinoma**	206(19.2%)	16(7.8%)	190(92.2%)		
**Smoking status**	**Former/current**	414(38.5%)	189(45.7%)	225(54.3%)	0.13	0.72
	**Never**	660(61.5%)	294(44.5%)	366(55.5%)		
**ECOG PS**	**0–1**	1058(98.5%)	474(44.8%)	584(55.2%)	0.84	0.36
	**2**	16(1.5%)	9(56.3%)	7(43.8%)		
**Repeat biopsy**	**Available**	343(31.9%)	230 (67.1%)	113(32.9%)	99.31	< 0.0001****
	**Unavailable**	731(68.1%)	253(34.6%)	478(65.4%)		
**SCLC transformation in re-biopsy**	**Transformation**	24(7.0%)	13(54.2%)	11(45.8%)	1.941	0.16
	**Non-transformation**	319(93.0%)	217(68.0%)	102(32.0%)		
**Driver gene mutation**						
**EGFR**	**Exon 18**	20(1.9%)				
	**Exon 19**	206(19.2%)				
	**Exon 20 excluding T790M**	22(2.0%)				
	**Exon 20 T790M**	18(1.7%)				
	**Exon 21**	244(22.7%)				
**ALK**		52(4.8%)				
**KRAS**		64(6.0%)				
**BRAF**		6(0.6%)				
**ROS1**		8(0.7%)				
**Co-mutation**	**EGFR/KRAS**	1(0.1%)				
	**EGFR/ALK**	5(0.5%)				
	**EGFR/KRAS/ROS1**	1(0.1%)				
	**EGFR/PI3K**	1(0.1%)				
	**ALK /KRAS**	2(0.2%)				
	**ALK /ROS1**	1(0.1%)				
	**KRAS /ROS1**	1(0.1%)				
**Wild type**	**all detected driver genes**	475(44.2%)				

**Table 2:** Clinical information of 24 NSCLC-to-SCLC transformation cases identified in this study

Group/Case	Sex/Age	Smoking	Clinical Stage	Initial pathology	Initial mutation	Treatment Before transformation	Time of TKI treatment before transformation	Re-biopsy specimen	Repeat mutation	Further exome sequencing
*EGFR*-mut/Case 1	F/59	Never	IV	LUAD	*EGFR*-19DEL	Chemo/TKI	10 months	Lung lesion	*EGFR*-19DEL	No
*EGFR*-mut/Case 2	F/50	Never	IV	LUAD	*EGFR*-19DEL	Chemo/TKI	12 months	Supraclavicular lymph nodes	*EGFR*-19DEL	No
*EGFR*-mut/Case 3	F/70	Never	IIIb	LUAD	*EGFR*-L858R	TKI	7 months	Lung lesion	*EGFR*-L858R	No
*EGFR*-mut/Case 4	M/50	Never	IV	LUAD	*EGFR*-L858R	Chemo/TKI	25 months	Lung lesion	*EGFR*-L858R/T790M	No
*EGFR*-mut/Case 5	F/66	Never	IV	LUAD	*EGFR*-19DEL	TKI	17 months	Supraclavicular lymph nodes	*EGFR*-19DEL	No
*EGFR*-mut/Case 6	M/63	Ever	IIIb	LUAD	*EGFR*-19DEL	TKI	23 months	Supraclavicular lymph nodes	*EGFR*-19DEL	No
*EGFR*-mut/Case 7	F/70	Never	IV	LUAD	*EGFR*-19DEL	TKI	16 months	Lung lesion	*EGFR*-19DEL	Yes
*EGFR*-mut/Case 8	F/56	Never	IIa	LUAD/operated	*EGFR*-19DEL	Surgical resection	/	Lung lesion	*EGFR*-19DEL	No
*EGFR*-mut/Case 9	M/82	Never	IIIa	LUAD/operated	*EGFR*-L858R	Surgical resection + TKI	24 months	Lung lesion	*EGFR*-L858R	No
*EGFR*-mut/Case 10	M/59	Never	IIa	LUAD/operated	*EGFR*-19DEL	Surgical resection	/	Lumbar metastases	*EGFR*-19DEL	No
*EGFR*-mut/Case 11	F/59	Never	IIa	LUAD/operated	*EGFR*-19DEL	Surgical resection	/	Lung lesion	*EGFR*-19DEL	Yes
*EGFR*-mut/Case 12	F/62	Never	IIb	LUAD/operated	*EGFR*-19DEL	Surgical resection + TKI	22months	Lung lesion	*EGFR*-19DEL	Yes
*EGFR*-mut/Case 13	M/69	Never	IIb	LUAD/operated	*EGFR*-G719C/S768I	Surgical resection	/	Lung lesion	*EGFR*-G719C/S768I	Yes
*EGFR*-wt/Case 14	F/44	Never	IV	LUAD	*ALK* rearrangement	Chemo	/	Lung lesion	Wt	No
*EGFR*-wt/Case 15	M/70	Never	IIIb	LUAD	*ALK* rearrangement	Chemo	/	Lung lesion	*ALK* rearrangement	Yes
*EGFR*-wt/Case 16	M/55	Never	IV	LUAD	Wt	Chemo	/	Lung lesion	Wt	No
*EGFR*-wt/Case 17	F/79	Ever	IIIb	LUAD	Wt	Chemo	/	Lung lesion	/	No
*EGFR*-wt/Case 18	F/68	Never	II	LUAD/operated	Wt	Surgical resection	/	Lung lesion	/	No
*EGFR*-wt/Case 19	M/64	Never	IIa	LUAD/operated	Wt	Surgical resection	/	Lung lesion	Wt	Yes
*EGFR*-wt/Case 20	M/60	Never	IIIa	LUAD/operated	Wt	Surgical resection	/	Lung lesion	Wt	Yes
*EGFR*-wt/Case 21	M/51	Never	IIb	LUAD/operated	Wt	Surgical resection	/	Lung lesion	Wt	Yes
*EGFR*-wt/Case 22	M/65	Ever	IIIa	LUSC/operated	Wt	Surgical resection	/	Mediastinal lymph node	/	No
*EGFR*-wt/Case 23	M/72	Ever	IIIa	LUSC/operated	Wt	Surgical resection	/	Lung lesion	/	No
*EGFR*-wt/Case 24	M/74	Ever	II	LUSC/operated	Wt	Surgical resection	/	Lung lesion	/	No

The median time to relapse as transformed SCLC in our study was 23.0 months (95%CI, 19.4–26.6) (Fig. 1D). After transformation, the mOS was 9.0 months (95%CI, 7.5–10.6), and the OS of *EGFR* wild type group was 4.0 months longer than that of *EGFR* mutant transformation cases (10.0 vs. 6.0 months, *P* = 0.016) (Fig. 1E). As typical cases presented above, transformed tumor tissues were positive for common NE markers such as Syn and CgA (Fig. 1B and C). We also dynamically monitored serum NSE, a common NE marker, and as expected, we found a significant serum NSE increasement when SCLC transformation occurred as compared to initial NSCLC diagnosis (34.37 ± 13.44 ng/ml vs. 16.03 ± 3.01 ng/ml, *P* < 0.0001) (Fig. 1F). In a typical case (Fig. 1G), serum NSE was always within the normal range at the initial diagnosis of LUAD and during the subsequent 5-month chemotherapy and 10-month Icotinib treatment, then a remarkable elevation was observed corresponding to the tumor progression on CT imaging when SCLC transformation was confirmed by re-biopsy. Furthermore, serum NSE decreased once SCLC therapy was administrated corresponding to a shrunk tumor on CT scan. A similar dynamic pattern of serum NSE level was also observed in the *EGFR* wildtype case who received surgery and multiline chemotherapies (Fig. 1H).

### Transformed SCLC harbored acquired gene alterations and *SMAD4* was associated with neuroendocrine phenotype in NSCLC under *TP53* inactivation

**Fig. 2:**
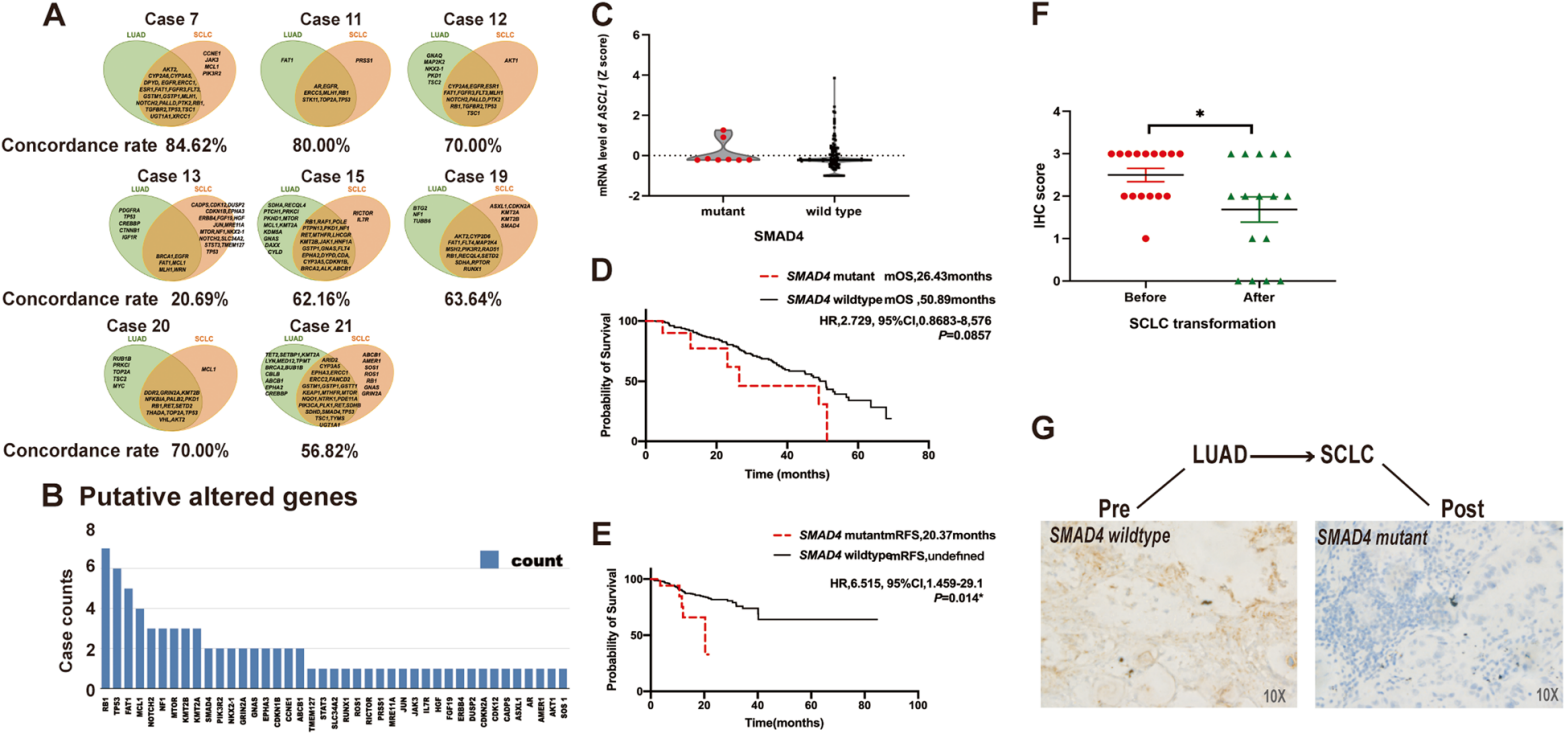
Sequencing results calculated from SCLC-transformed cases and SMAD4 was associated with neuroendocrine phenotype in NSCLC-to-SCLC transformation.** A** A high percentage of shared mutant genes was identified in each transformed case. The ratio of shared altered genes in each transformed case was 84.62%, 80.00%, 70.00%, 20.69%, 62.16%, 63.64%, 70.00% and 56.82% in Case 7, 11, 12, 13, 15, 19, 20 and 21, respectively. **B** The mutant rate of genes altered specifically in transformed SCLCs. 41 genes were found specifically altered in transformed SCLC tumors as sorted by mutation rate. Genes with a mutant rate of more than 25% included *RB1, TP53, FAT1, MCL1, NOTCH2, NF1, MTOR, KMT2B, KMT2A, SMAD4, PIK3R2, NKX2-1, GRIN2A, GNAS, EPHA3, CDKN1B, CCNE1* and *ABCB1*. **C**, *SMAD4* mutation was associated with mRNA level of neuroendocrine genes in NSCLC patients based on TCGA database. The data was determined as described in note of Table 3. **D-E** *SMAD4* mutation was an independent factor of poor overall survival (**D**) and relapse-free survival (**E**) of NSCLC drawn from TCGA database. These 13 studies as described in note of Table 3 included a total of 2847 patients. Of patients with available sequencing results for their tumor samples, 1503 were followed for overall survival and 340 were followed for relapse-free survival. These patients were grouped according to mutation status in *SMAD4, TP53* and *EGFR.* Overall survival of patients with co-mutations of *SMAD4, TP53* and *EGFR* compared to patients with co-mutations of *TP53* and *EGFR*. Relapse-free survival in patients with *SMAD4* and *TP53* co-mutations compared to patients with wild-type *SMAD4, TP53* and *EGFR*. The Kaplan Meier method was used for all single-factor survival analyses. **F** SMAD4 expression was decreased in transformed SCLCs. Among 24 transformation cases, we obtained pre- and post-transformation slices for SMAD4 staining from 16 cases. The average IHC score of SMAD4 staining was 2.5 in primary NSCLCs and was 1.7 in transformed SCLCs (*P* = 0.027). **G** SMAD4 mutation led to loss of SMAD4 protein expression in representative transformation case. In transformed case 19, *SMAD4* was wildtype in primary LUAD tissue and was strongly positive by IHC staining. And in transformed SCLC tumor, *SMAD4* was mutant in transformed SCLC (p.Q289X) and was negative in IHC staining

Among 24 transformation cases, only 8 cases had sufficient tumor tissues before and after transformation for further sequencing covering 416 cancer-related genes (Table S1). Totally, we identified 81 altered genes with 107 mutations (Table S2) and observed a high percentage of shared gene mutations in each case (Fig. 2A). It’s widely known that inheriting *EGFR* mutations is an evidence of SCLC transformation, which was also identified in our cases (Table 2). Besides, for SCLC transformation cases from *EGFR* wildtype LUADs, we identified the inheritance of *RB1* and/or *TP53* alterations in transformed SCLCs, such as *TP53* R158L/E271X in Case 15, *RB1* K294X in Case 19 (Table S3).

**Table 3:** SMAD4 mutation was associated with ASCL1 expression especially in TP53-mutant NSCLC calculated from TCGA database

		All cases (*n* = 168)			*TP53* mutant (*n* = 87)			*TP53* wildtype (*n* = 81)		
		*SMAD4* mutant	*SMAD4* wildtype	*P* value	*SMAD4* mutant	*SMAD4* wildtype	*P* value	*SMAD4* mutant	*SMAD4* wildtype	*P* value
***ASCL1*** **Expression level**	**High**	75.0% (6/8)	34.4% (55/160)	0.027*	100.0%(3/3)	28.6%(24/84)	0.028*	60.0%(3/5)	40.8%(31/76)	0.645
	**Low**	25.0% (2/8)	65.6% (105/160)		0.0%(0/3)	71.4%(60/84)		40.0%(2/5)	59.2%(45/76)	

The data was taken from The Cancer Genome Atlas (TCGA) for our bioinformatics analysis. We selected 13 NSCLC studies with patient follow-up data and gene mutation status in TCGA, which were Lung Adenocarcinoma (MSKCC, 2021), Non-Small Cell Lung Cancer (University of Turin, Lung Cancer 2017), Lung Adenocarcinoma (OncoSG, Nat Genet 2020), Non-Small Cell Lung Cancer (MSKCC, J Clin Oncol 2018), Lung Adenocarcinoma (Broad, Cell 2012), Non-Small Cell Lung Cancer (MSK, Cancer Cell 2018), Non-Small Cell Lung Cancer (TRACERx, NEJM & Nature 2017), Lung Adenocarcinoma (TCGA, PanCancer Atlas), Lung Adenocarcinoma (NPJ Precision Oncology, MSK 2021), Lung Adenocarcinoma (MSKCC, 2020), Lung Adenocarcinoma (TSP, Nature 2008), Non-small cell lung cancer (MSK, Science 2015) and Lung Adenocarcinoma (MSKCC, Science 2015). RNA expression data were obtained from the two studies of Lung Adenocarcinoma (TCGA, PanCancer Atlas) and Non-Small Cell Lung Cancer (TRACERx, NEJM & Nature 2017). 168 patients with EGFR mutations in these two studies were analyzed by us for correlation of neuroendocrine marker expression with SMAD4 mutations. After conversion to z-score, RNA expression data from both studies were integrated. The chi-square test was used for correlation analysis. Note: * *P*<0.05

We also identified 41 genes which were mutated specifically in transformed SCLC tumors, and 18 genes showed a mutant rate of more than 25% in 8 sequenced cases such as *RB1, TP53, NOTCH2, SMAD4, PIK3R2* and *EPHA3* (Fig. 2B). We analyzed the associations between these genes and NE related genes expression in NSCLC based on TCGA database, and in *SMAD4*-mutant LUADs, the ratio of *ASCL1-*upregulated LUADs was higher than that in SMAD4-wildtype cases (75.0% vs. 34.4%, *P* = 0.027) especially under *TP53* inactivation (Fig. 2C; Table 3). We also conducted pathway enrichment analysis and found that *SMAD4* was involved in multiple pathways including cell size regulation and also neuron differentiation (Fig. S1B). Hence, we inferred that *SMAD4* contributed to small-cell transition, especially by affecting the neuroendocrine phenotype under *TP53* inactivation. We detected Smad4 expression in 16 small-cell transformed cases by IHC, and observed a significantly decrease of Smad4 expression in transformed SCLC specimen (IHC score: 2.5 vs. 1.7, *P* = 0.027) (Fig. 2F). In transformed cases, *SMAD4* mutation was an inactivation alteration because no protein expression was detected in *SMAD4* mutant SCLC specimen (Fig. 2G).

### *SMAD4* deletion promoted tumor growth and induced multi-drug resistance in *TP53* -inactivated NSCLC

To further explore the gene function, we knocked out *SMAD4* in *TP53*-inactivated HCC827 and A549-TP53^-/-^ cells and we further knocked out *RB1* due to its essential role in SCLC formation. As shown in Fig. S2, we screened several clones labeled as HCC827-SMAD4^-/-^, HCC827-SMAD4^-/-^RB1^-/-^, A549-TP53^-/-^-SMAD4^-/-^ and A549-TP53^-/-^-SMAD4^-/-^RB1^-/-^ in which no Smad4 and/or Rb1 expression was detected.

**Fig. 3:**
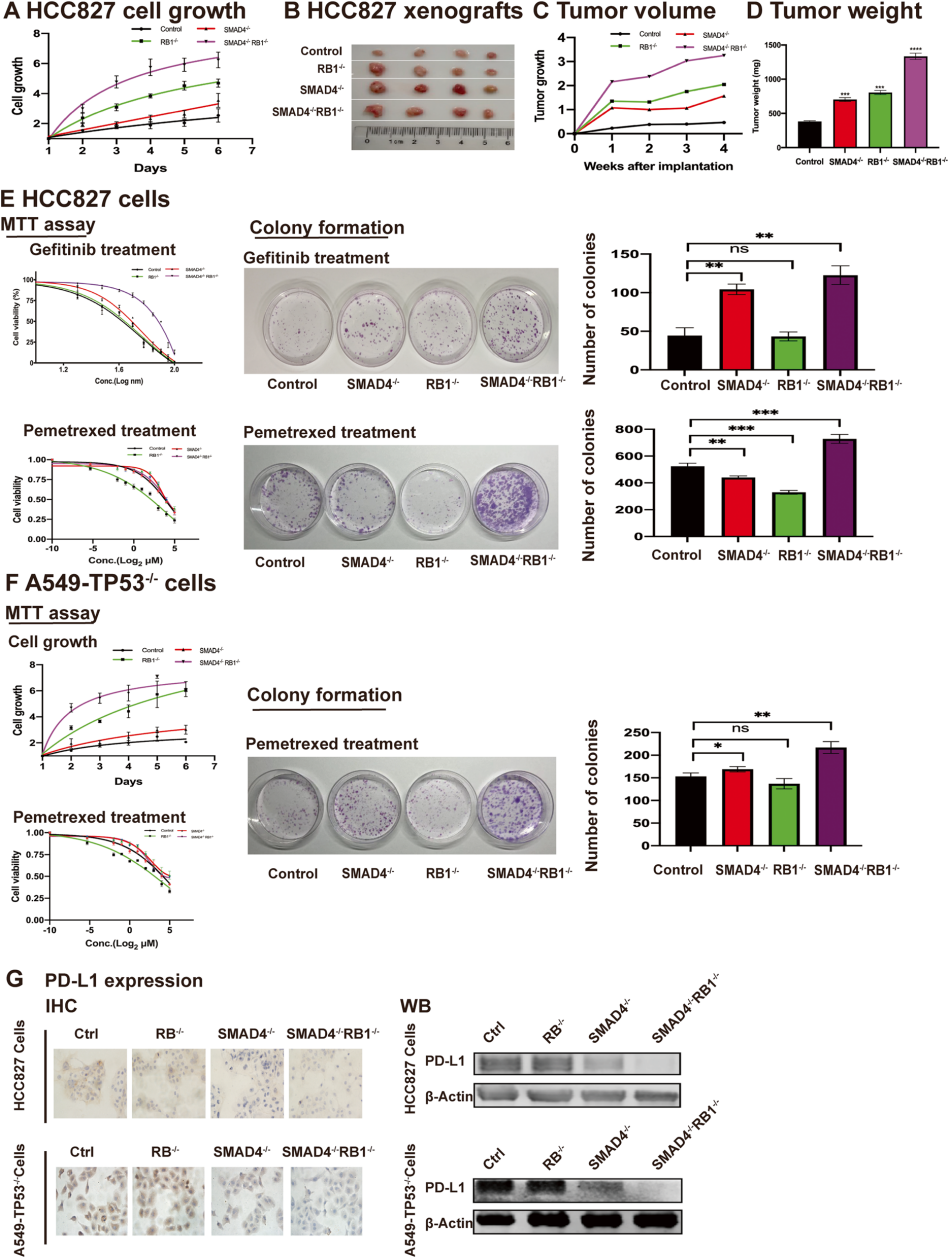
SMAD4 loss led to cell growth and multiple drug resistance in TP53-deficient NSCLC. HCC827 cells were naturally *TP53* inactivated (c.652_654delGTG) and A549-TP53^-/-^ was manually knocked out *TP53* previously in our lab. **A** SMAD4 loss led to cell growthin vitro. As compared to the control group, the cell growth of HCC827-SMAD4^-/-^ was significantly promoted, and inactivation or co-inactivation of RB1 further enhanced the cell growth ability. **B**,** C** and **D**, SMAD4 loss led to cell growthin vivo. In xenografts, the tumor volume and tumor weight were significantly increased when *SMAD4* was knocked out, which were further enhanced when *RB1* was co-inactivated. **E** SMAD4 loss led to drug resistance of Gefitinib and Pemetrexed in HCC827 cells by MTT assay and colony formation. *EGFR*-mutant HCC827 cells were treated with Gefitinib, the 1st generation EGFR-TKI, the IC50 of control group was 56.76nM and was 57.08nM in SMAD4^-/-^ cells. The IC50 of RB1^-/-^ cells was 56.98nM, which was similar to that of control group and the IC50 of SMAD4^-/-^RB1^-/-^ cells was 211.70nM (E, MTT assay, Gefitinib treatment). In colony formation assay, the numbers of colonies were 44 ± 10, 43 ± 6, 104 ± 6, 123 ± 12 of control group, RB1^-/-^ (*P* = 0.8894), SMAD4^-/-^ (*P* = 0.0011) and SMAD4^-/-^RB1^-/-^ cells (*P* = 0.0010), respectively (E, colony formation, Gefitinib treatment). HCC827 cells were treated with Pemetrexed, the IC50 of control group was 10.06µM and was 19.5µM in SMAD4^-/-^ cells. The IC50 of RB1^-/-^ cells was 2.69µM and the IC50 of SMAD4^-/-^RB1^-/-^ cells was 21.10µM (E, MTT assay, Pemetrexed treatment). In colony formation assay, the numbers of colonies were 524 ± 23, 440 ± 11,330 ± 14, 728 ± 33 of control group, SMAD4^-/-^ (*P* = 0.0047), RB1^-/-^ (*P* = 0.0002), and SMAD4^-/-^RB1^-/-^ cells (*P* = 0.0009), respectively (E, colony formation, Pemetrexed treatment). **F** SMAD4 loss led to cell growth and drug resistance of Pemetrexed in A549 cells by MTT assay and colony formation. As compared to the control group, the cell growth of A549-TP53^-/-^SMAD4^-/-^ was significantly promoted, and inactivation or co-inactivation of *RB1* further enhanced the cell growth ability. In Pemetrexed treatment, the IC50 of control group was 5.63µM and was 6.02µM in SMAD4^-/-^ cells. The IC50 of RB1^-/-^ cells was 2.67µM and the IC50 of SMAD4^-/-^RB1^-/-^ cells was 5.57µM (F, MTT assay, Pemetrexed treatment). In colony formation assay, the numbers of colonies were 153 ± 7, 169 ± 6, 137 ± 11, 217 ± 13 of control group, SMAD4^-/-^ (*P* = 0.0491), RB1^-/-^ (*P* = 0.1076), and SMAD4^-/-^RB1^-/-^ cells (*P* = 0.0019), respectively (F, colony formation, Pemetrexed treatment). **G** SMAD4 loss led to down-regulated expression of PD-L1 in NSCLC cells. The expression of PD-L1was decreased when SMAD4 was lost in HCC827 and A549-TP53^-/-^ cells by IHC and WB assay. No obvious changes of PD-L1 expression were found when *RB1* was lost alone. Abbreviation: ns, no significance

We then explored the effect of Smad4 loss on cell growth. As a result, Smad4 deletion significantly promoted the HCC827 cell growth as compared to the control group, suggesting the tumor suppressor role of *SMAD4*. As expected, inactivation or co-inactivation of *RB1* further enhanced the cell growth ability (Fig. 3A). In in vivo experiments, we also observed the significantly increased tumor growth and tumor weight when *SMAD4* was knocked out (Fig. 3B, C and D), which was consistent with the results of in vitro experiment.

We treated EGFR-mutant HCC827 cells with Gefitinib, the 1st generation EGFR-TKI, and accordingly, the IC50 didn’t show significant changes when *SMAD4* or *RB1* was knocked out alone as compared to control group by MTT assay (57.08nM, 56.98nM vs. 56.76nM, *P* > 0.05). However, the IC50 was significantly increased when *SMAD4* and *RB1* were co-inactivated as compared to control group (211.70 nM vs. 56.76nM, *P* > 0.05). We also treated the cells with Pemetrexed, the most recommended regimen as first-line chemotherapy of LUAD. Consequently, Smad4 loss led to the significantly increased IC50 in HCC827 cells (SMAD4^−/−^, 19.5µM vs. Control, 10.06µM, *P* = 0.0176). As compared to control group, the IC50 was significantly decreased when *RB1* was knocked out alone (10.06µM vs. 2.69 µM, *P* = 0.0003). However, if *SMAD4* was co-inactivated, the sensitivity of cells to pemetrexed decreased further (SMAD4^−/−^RB1^−/−^, 21.10µM vs. Control, 10.06µM, *P* = 0.0057) (Fig. 3E). Similar effect of Smad4 on cell growth was achieved in A549-TP53^−/−^ cells while no obvious change of Pemetrexed sensitivity was observed (Fig. 3F).

In addition, we detected the expression of PD-L1. Consequently, PD-L1 was down-expressed in SMAD4^−/−^ cells while no obvious decrease was observed in RB1^−/−^ cells as compared to control group by IHC staining and western blotting, which indicates the unsatisfactory treatment efficiency of PD-1/PD-L1 antibody therapy (Fig. 3G).

### *SMAD4 *loss induced acquired neuroendocrine phenotype independently of *RB1* status by upregulating *ASCL1* transcription in vitro and in vivo

**Fig. 4:**
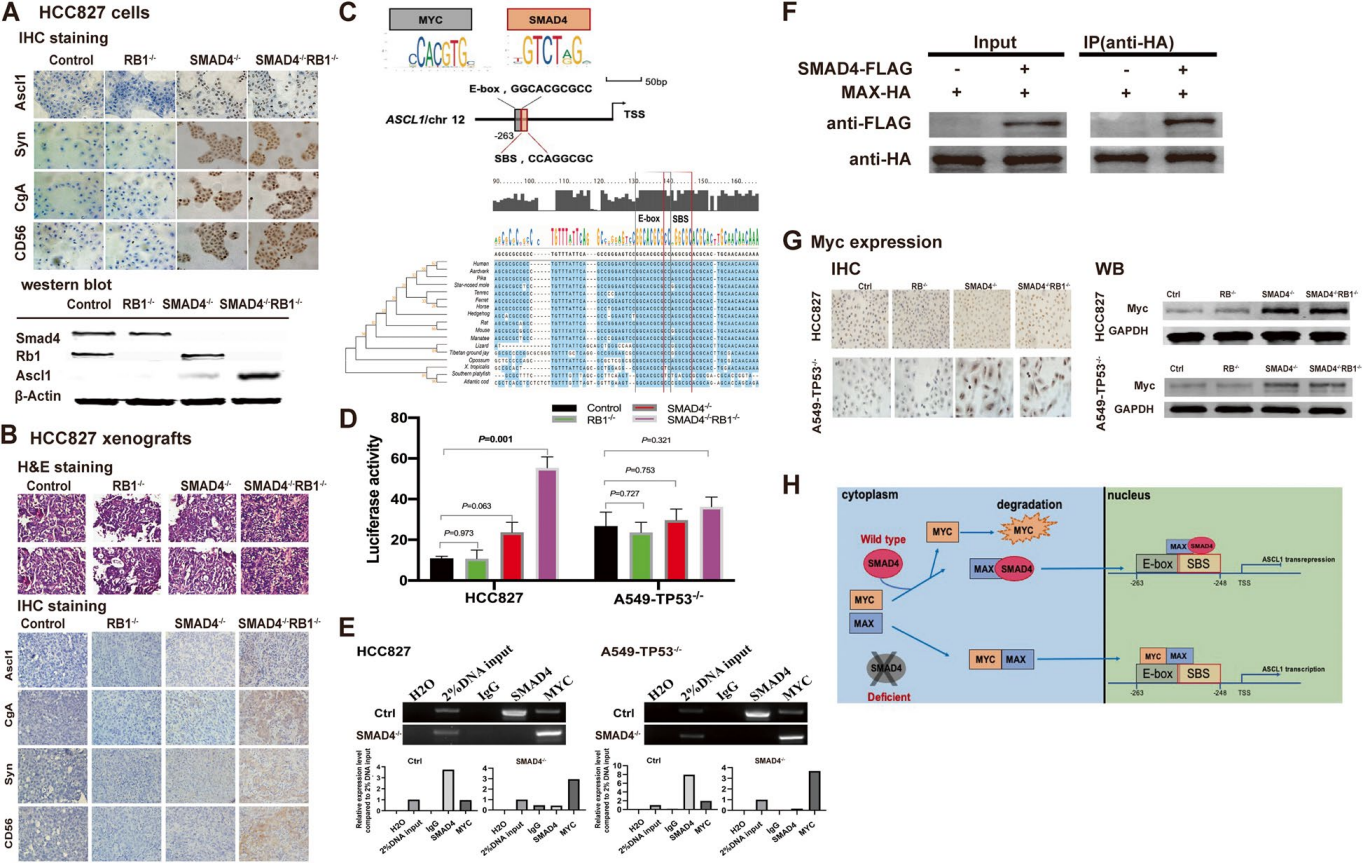
SMAD4 loss induced neuroendocrine phenotype independently of *RB1* by regulating ASCL1 expression in TP53-deficient NSCLC. **A** SMAD4 loss induced neuroendocrine phenotype independently of RB1 in vitro. By IHC staining, positive expression of neuroendocrine markers Ascl1, Syn (Synaptophysin), CgA (ChromograninA) and CD56 (also known as NCAM) was identified in SMAD4-deficient HCC827 cells, and no obvious changes of these mentioned markers were found when *RB1* was knocked out alone. In western blotting, upregulated Ascl1 was identified in SMAD4-deficient lane, which was strongest in *SMAD4* and *RB1* co-inactivated lane, while no obvious change was found in RB1-deficient lane as compared to control. **B** SMAD4 loss induced neuroendocrine phenotype independentlyof *RB1 in vivo****.*** HCC827 xenografts were established and tumors were resected for further H&E and IHC staining. The SMAD4 deficient tumors showed more cells with a finely granular (“salt and pepper”) chromatin pattern and less with scant cytoplasm. with positive expression of neuroendocrine markers Ascl1, CgA, Syn and CD56, which were stronger when *RB1* was co-inactivated. While no obvious changes of morphology or neuroendocrine phenotype were observed when *RB1* was knocked out alone in HCC827 xenografts. **C** Transcriptional binding site analysis and motif enrichment analysis. The transcriptional binding site analysis was performed on http://jaspar.genereg.net/ and the motif enrichment analysis was performed on Clustal W. E-box, which is the MYC-binding site and SMAD4-binding site (SBS) were identified in -1000 upstream area of the human *ASCL1* gene and were highly conservative in different species, which indicated that SMAD4 can directly bind to the transcriptional binding site and regulate transcription of *ASCL1.* And the base overlap between SBS and E-box indicates the potential competition in *ASCL1* transcriptional binding region. **D** SMAD4 loss upregulated ASCL1 transcription level in NSCLC cells. By luciferase assay, *ASCL1* promoter plasmids were transfected in each group of HCC827 and A549-TP53^-/-^ cells. The average luciferase activity in HCC827 cells was 10.88, 10.73, 31.36 and 55.40 of control group, RB1^-/-^ (*P* = 0.9728), SMAD4^-/-^ (*P* = 0.0008), and SMAD4^-/-^RB1^-/-^ cells (*P* = 0.0012), respectively. The average luciferase activity in A549-TP53^-/-^ cells was 20.11, 20.27, 31.40 and 36.24 of control group, RB1^-/-^ (*P* = 0.9496), SMAD4^-/-^ (*P* = 0.0620), and SMAD4^-/-^RB1^-/-^ cells (*P* = 0.0323), respectively. **E** SMAD4 and Myc competitively regulate *ASCL1*transcription. By ChIP assay, we found that in SMAD4-wildtype NSCLC cells, SMAD4, instead of Myc, bound to *ASCL1*; while when SMAD4 was deficient, MYC would bind to *ASCL1* to regulate transcription. **F **SMAD4 interacts with MAX in HCC827 cells transfected with MAX-HA and SMAD4-FLAG. **G** SMAD4 loss led to upregulated expression of Myc in NSCLC cells. The expression of Myc was increased when SMAD4 was lost in HCC827 and A549-TP53^-/-^ cells by IHC and western blotting assay. No obvious changes of Myc expression were found when *RB1* was lost alone. **H** The putative mechanism of SMAD4-mediated neuroendocrine phenotype acquisition in NSCLC cells. Note: ns, no significance; *, *P* < 0.05; **, *P* < 0.01; ***, *P* < 0.001

We detected NE markers in HCC827 cells and confirmed the positive expression of Syn and also ASCL1, the upstream regulator of multiple NE markers in SCLC when *SMAD4* was knocked out. No obvious change of neuroendocrine makers expression was detected when *RB1* was knocked out only (Fig. 4A). Similar results were also observed in A549-TP53^-/-^ cells (Fig. S3A). In murine xenografts, we found scattered CgA- and Syn-positive lesions in SMAD4^-/-^ xenografts, and highly stained CgA, Syn and CD56 in SMAD4^-/-^ RB1^-/-^xenografts by IHC staining (Fig. 4B, IHC staining). SMAD4 is an important transcriptive factor (TF) regulating gene transcription. By motif analysis, we found Smad4 binding site (SBS) in the promoter region of *ASCL1* gene, which has a base overlap with the E-box region, the binding site of MYC (Fig. 4C). We further transfected established cell models with *ASCL1* luciferase reporter vectors and we demonstrated the significant increasement of *ASCL1* transcriptional activity in HCC827-SMAD4^-/-^RB1^-/-^ cells (*P* = 0.001, Fig. 4D), while no obvious difference of activity was observed when *RB1* was knocked out only. We further revealed the negative regulatory effect of SMAD4 on *ASCL1* transcription by ChIP in *SMAD4*-wildtype NSCLC cells (Fig. 4E). MYC binds gene promoters and activate transcription by forming a heterodimer with its primary partner MAX [12]. We also idenfied the interaction between SMAD4 and MAX in HCC827 cells by Co-IP (Fig. 4F).

Therefore, we proposed the hypothesis that SMAD4 can compete with MYC for MAX to form transcriptional complexes to regulate *ASCL1* transcription. In *SMAD4*-wildtype NSCLC, *ASCL1* transcription is suppressed by MAX/SMAD4 complex, and MYC degradation is accelerated due to instability of protein without complex formation. When *SMAD4* is loss-of-function mutant, *ASCL1* transcription is activated by MYC/MAX complex, which will further regulate neuroendocrine phenotype in NSCLC (Fig. 4H).

### Myc inhibitor acts as the potential therapy for NSCLC with acquired neuroendocrine phenotype mediated by SMAD4 deficiency

**Fig. 5:**
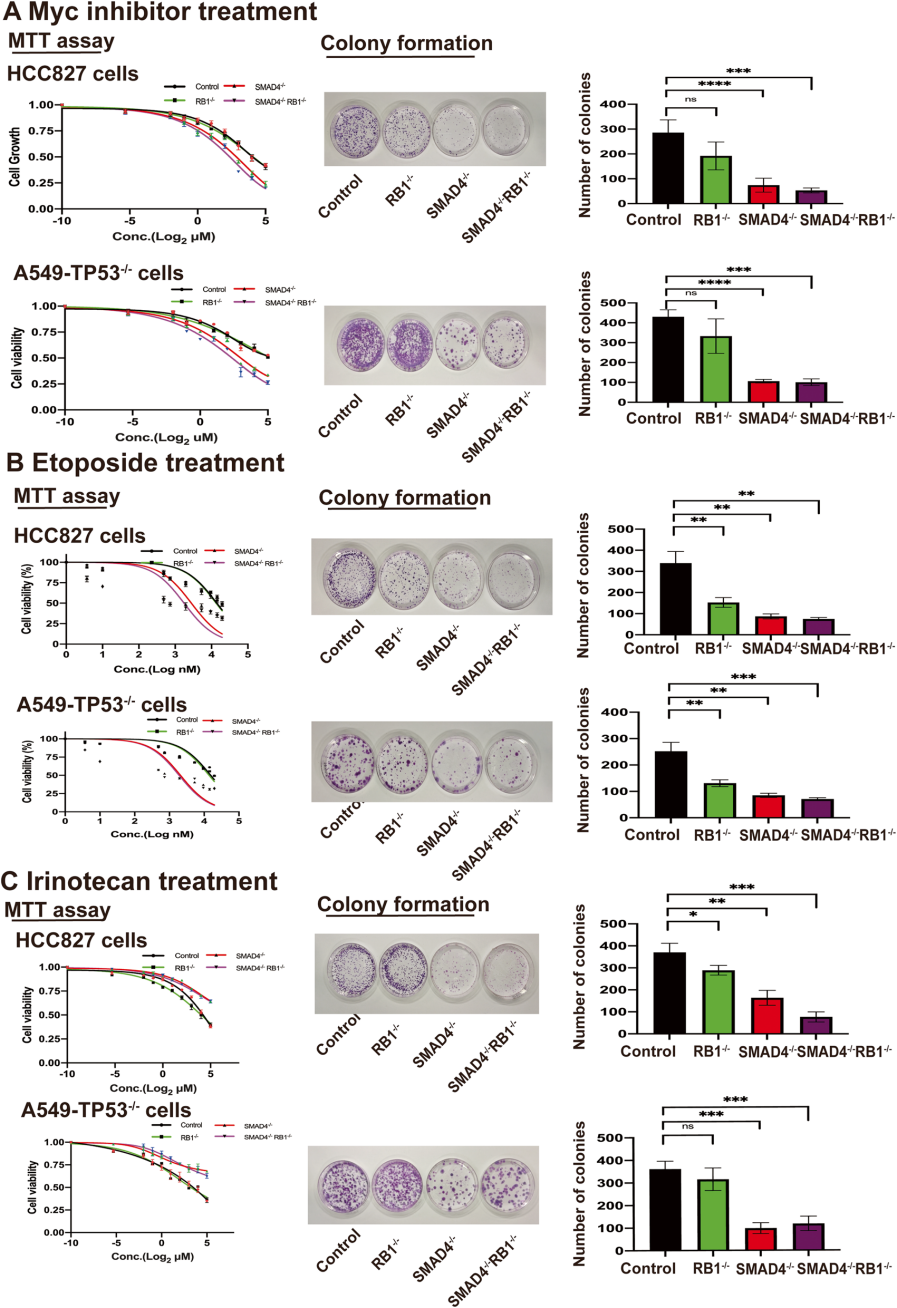
Treatment in SMAD4-loss mediated resistant NSCLC cells. **A** SMAD4 loss increased sensitivity of NSCLC cells to Myc inhibitor. HCC827 and A549-TP53^−/−^ cells were treated with Myc inhibitor. In MTT assay, the IC50 of HCC827 cells was 7.65µM, 5.51µM, 5.97µM, and 4.08µM of control group, SMAD4^−/−^, RB1^−/−^, and SMAD4^−/−^RB1^−/−^ cells, respectively. The IC50 of A549-TP53^−/−^ cells was 4.13µM, 3.61µM, 3.08µM, and 2.74µM of control group, SMAD4^−/−^, RB1^−/−^, and SMAD4^−/−^RB1^−/−^ cells, respectively. In colony formation assay, the numbers of HCC827 colonies were 286 ± 51, 74 ± 28,192 ± 56, 53 ± 10 of control group, SMAD4^−/−^ (*P* < 0.0001), RB1^−/−^ (*P* = 0.1454), and SMAD4^−/−^RB1^−/−^ cells (*P* = 0.0001), respectively. In colony formation assay, the numbers of A549-TP53^−/−^ colonies were 430 ± 35, 106 ± 9,333 ± 87, 101 ± 17 of control group, SMAD4^−/−^ (*P* = 0.0004), RB1^−/−^ (*P* = 0.2567), and SMAD4^−/−^RB1^−/−^ cells (*P* = 0.0009), respectively. **B** SMAD4 loss increased Etoposide sensitivity in NSCLC cells. HCC827 and A549-TP53^−/−^ cells were treated with etoposide. In MTT assay, the IC50 of HCC827 cells was 13.97µM, 2.79µM, 13.41µM, and 1.75µM of control group, SMAD4^−/−^, RB1^−/−^, and SMAD4^−/−^RB1^−/−^ cells, respectively. The IC50 of A549-TP53^−/−^ cells was 15.43µM, 2.08µM, 13.85µM, and 1.90µM of control group, SMAD4^−/−^, RB1^−/−^, and SMAD4^−/−^RB1^−/−^ cells, respectively. In colony formation assay, the numbers of HCC827 colonies were 339 ± 55, 87 ± 12,153 ± 23, 75 ± 7 of control group, SMAD4^−/−^ (*P* = 0.0015), RB1^−/−^ (*P* = 0.0059), and SMAD4^−/−^RB1^−/−^ cells (*P* = 0.0012), respectively. In colony formation assay, the numbers of A549-TP53^−/−^ colonies were 252 ± 34, 85 ± 8,131 ± 13, 71 ± 5 of control group, SMAD4^−/−^ (*P* = 0.0011), RB1^−/−^ (*P* = 0.0043), and SMAD4^−/−^RB1^−/−^ cells (*P* = 0.0008), respectively. **C** SMAD4 loss had no obvious effect on Irinotecan sensitivity in NSCLC cells. HCC827 and A549-TP53^−/−^ cells were treated with ironotican. In MTT assay, the IC50 of HCC827 cells was 14.38µM, 8.96µM, 4.80µM, and 5.82µM of control group, SMAD4^−/−^, RB1^−/−^, and SMAD4^−/−^RB1^−/−^ cells, respectively. The IC50 of A549-TP53^−/−^ cells was 1.84µM, 0.87µM, 1.48µM, and 2.19µM of control group, SMAD4^−/−^, RB1^−/−^, and SMAD4^−/−^RB1^−/−^ cells, respectively. In colony formation assay, the numbers of HCC827 colonies were 371 ± 41, 164 ± 34,289 ± 22, 77 ± 23 of control group, SMAD4^−/−^ (*P* = 0.0025), RB1^−/−^ (*P* = 0.0379), and SMAD4^−/−^RB1^−/−^ cells (*P* = 0.0004), respectively. In colony formation assay, the numbers of A549-TP53^−/−^ colonies were 361 ± 35, 98 ± 24,315 ± 50, 118 ± 32 of control group, SMAD4^−/−^ (*P* = 0.0004), RB1^−/−^ (*P* = 0.2567), and SMAD4^−/−^RB1^−/−^ cells (*P* = 0.0009), respectively

As mentioned above, Myc was upregulated when *SMAD4* was inactivated (Fig. 4G). We treated each group with a pan-Myc inhibitor, and the result showed that the IC50 of HCC827-SMAD4^−/−^RB1^−/−^ was significantly decreased as compared to control group (4.08 µM vs. 7.65 µM, *P* = 0.0075). Similar result was observed in A549-TP53^−/−^SMAD4^−/−^RB1^−/−^ group although there was no statistical significance (2.74 µM vs. 4.13 µM, *P* = 0.1776) (Fig. 5A). Besides Myc inhibitor, Bcl2 and DLL-3 [13] antibodies are also potential targeted therapy targets for SCLC. We also detected the expression of Bcl2 and DLL-3 in established cell models. As a result, we found no obvious changes of Bcl2 and DLL-3 expression (Fig. S3E).

Chemotherapy still serves as the mainstream therapies for high-grade lung neuroendocrine tumors, which mainly refer to SCLC. We treated each group of cells with etoposide and irinotecan. As compared to control group, the IC50 of Etoposide was significantly decreased in HCC827-SMAD4^−/−^ (14.0 µM vs. 2.79 µM, *P* < 0.0001) and HCC827-SMAD4^−/−^RB1^−/−^ (14.0 µM vs. 1.75 µM, *P* < 0.0001). Knockout of *RB1* alone had no significant effect on Etoposide sensitivity as compared to control group (14.0 µM vs. 13.4 µM, *P* = 0.8007) and similar results were observed in A549-TP53^−/−^ group (Fig. 5B). No obvious changes of irinotecan sensitivity were determined when *SMAD4* and/or *RB1* was deficient (Fig. 5C).

## Discussion

SCLC transformation is usually identified by re-biopsy at the time of relapse or disease progression [14–16].Recent reports [10, 11] demonstrated the rapid deterioration and shorter OS of transformed cases, which strongly requests urgent and more comprehensive understandings of this phenomenon. Here, our work indicates that SCLC transformation occurs regardless of *EGFR* mutation status/TKIs treatment and pathological type in NSCLC population, and also puts forward a set of potential genes contributing to the histological transformation.

To the best of our knowledge, this is the first time that an incidence of transformation in the *EGFR* wild type NSCLCs was reported. The majority of previous studies focused on *EGFR* mutant patients, only several individual cases reported the SCLC transformation from *EGFR* wild-type NSCLC [3, 17–19]. In our cohort consisting of 591 *EGFR* wild type patients, 11 transformed cases were identified in 113 individuals with repeat biopsy (11/113, 9.73%). The incidence rate in the *EGFR* mutant group is 5.65% (13/230), which is similar to previous study [20] involving 155 TKIs-resistant patients. Actually, in our work, the re-biopsy was recommended to some of the *EGFR* wild type patients by our clinicians based on their dynamic serum NSE change and disease progression, which may cause the data bias because of the intentional selection to avoid excessive medical examination. Moreover, this result also emphasizes the value of serum NSE monitoring in SCLC transformation as the changing curve shown in Fig. 1G, H.

It is highly necessary to excavate the genetic predictors of pathological evolution towards SCLC. As previously studied [7, 21], the concurrent *RB1* and *TP53* alterations of initial LUAD tumors at the early-stage lead to a higher tendency to SCLC transformation. Correspondingly, our result also showed a higher mutant rate of *RB1* and *TP53* in funder LUAD tumors. Besides, we also found the inherited *TP53* and/or *RB1* mutations in transformed SCLCs from *EGFR* wildtype cases, which further supported the occurrence of SCLC transformation in *EGFR* wildtype cohort. By analyzing the specific altered genes in transformed SCLC tumors, we revealed a set of candidate genes, such as *RB1, AKT1, SMAD4*, and et al. Work by Park J.W. et al. [22] suggests a distinguished role of *AKT1*, besides *TP53* and *RB1*, in small cell prostate cancer evolution. Previously, Smad4 was reported to be an independent factor of prognosis in NSCLC [23]. While the relationship between *SMAD4* and NE phenotype is rarely reported in NSCLC. Nicky et al. [24] reported *SMAD4* mutation in transformed SCLC patient while no further validation was conducted. Our work demonstrated that *SMAD4* can promote the aggressive tumor behavior and induce NE phenotype and EGFR-TKI and pemetrexed resistance independently of *RB1* status for the first time. Ascl1 is required for SCLC formation [25] ,our work also revealed that *SMAD4* deficiency can upregulate *ASCL1* transcription inducing NE phenotype in NSCLC. Consistent to our hypothesis [26], *MYC* amplification promoted tumor development with low Ascl1 expression though in SCLC mouse models.

For now, platinum-based etoposide still serves as the first choice for transformed SCLCs with nearly 50% [10, 11] clinical response rates. With accessible follow-up data in our study, the clinical response rate was 57.1% (8/14) in 14 transformed patients treated with platinum-etoposide. It’s a tough task to find target therapy for transformed SCLCs, as well as for primary SCLCs. In our study, we found the upregulated expression of Myc and an increased sensitivity to Myc inhibitor when SMAD4 was deficient. Moreover, the prognosis was significantly shortened for NSCLC patients with acquired NSE increasement during disease course, whatever SCLC transformation occurs or not (Fig. S2G). Thus, we inferred that SMAD4 deficiency led to drug resistance partially by inducing NE phenotype and Myc inhibitor could be the potential targeted therapy in NSCLC.

There are still several limitations and remaining questions in our work. Firstly, we just obtained 8 paired samples for sequencing. It will be more sufficient if more cases could be sequenced. Nevertheless, considering the low occurrence rate and the tiny repeat biopsy specimens, it’s hard to receive abundant samples from transformation cases. Secondly, we just explored the function of *SMAD4*, and the contributions of other genes remain to be studied because it is highly possible that the transformation of SCLC requires the involvement of multiple genes.

## Conclusions

To conclude, our results provide new insights into SCLC transformation from NSCLC, especially fill the gap in *EGFR* wide type cohort. SCLC transformation is a common phenomenon in NSCLC individuals, which is irrespective of *EGFR* mutation status, treatment regimens, and pathological subtype. In our study, we suggested that *SMAD4* mutation can promote SCLC transformation and further lead to drug resistance in NSCLC. And the subsequent strategy is much more important from a clinical point of view. Our study proposed the potential therapeutic effect of Myc inhibitor besides traditional etoposide and iritecan. Some recent studies [27] also emphasized the potential of Myc inhibitors in SCLC treatment, which requires more study to support our view of Myc inhibitor for the treatment of transformed SCLC. Clinicians need to be highly aware of this phenomenon, and more in-depth researches are in pressing need.

### Supplementary Information

**Additional file 1.**

## Data Availability

All data are available in the main text or the supplementary materials.
