# Supplementary material for: Transformation to small cell lung cancer is irrespective of EGFR and accelerated by SMAD4-mediated ASCL1 transcription independently of RB1 in non-small cell lung cancer
Source: Cell Commun Signal. 2024 Jan 17;22:45. doi: 10.1186/s12964-023-01260-8 (PMC10795321; doi:10.1186/s12964-023-01260-8)
Supplement: Supplementary file 2 — Additional file 1. [file 12964_2023_1260_MOESM1_ESM.docx]

Supplementary Text

Antibodies used in immunohistochemistry staining and western blotting and regimens used in drug sensitivity detection

Antibodies used in IHC staining were as follows: pan-cytokeratin (CK, AE1/AE3, DAKO), p63 (DAK-p63, DAKO), chromogranin A (CgA, LK2H10, DAKO), synaptophysin (Syn, OTI1C9, DAKO), neural cell adhesion molecule (CD56/NCAM, UMAB83, DAKO), Vimentin (Vim, clone V9, DAKO), TTF-1 (8G7G3/1, DAKO), Ki-67, MIB-5, DAKO), Napsin A (MA5-27040, Thermofisher), P40 (Art. No. PA5-28477, Thermofisher), Ascl1(D-7, sc-374104, Santa cruz), Smad4 (D3R4N, #46535, Cell signaling technology) and Myc (#2276, Cell signaling technology).

Antibodies used in WB were as follows: Smad4 (D3R4N, #46535, Cell signaling technology), Rb1(PA5-99502, Thermofisher), Ascl1(D-7, sc-374104, Santa cruz), PD-L1(22C3, Dako M3653), DLL3(#78110, Cell signaling technology), Bcl2(124, #15071, Cell signaling technology), Myc (#2276, Cell signaling technology), β-Actin (ab8227, abcam) and GAPDH (ab181602, abcam).

Regimens used in drug sensitivity detection were as follows: MTT, Etoposide (CAS No. 33419-42-0, MedChemExpress), Irinotecan (CAS No. 97682-44-5, MedChemExpress), pemetrexed (CAS No. 137281-23-3, MedChemExpress), Gefitinib (ZD1839, CAS No. 184475-35-2, Selleck), Myc inhibitor (MCE HY-129600).


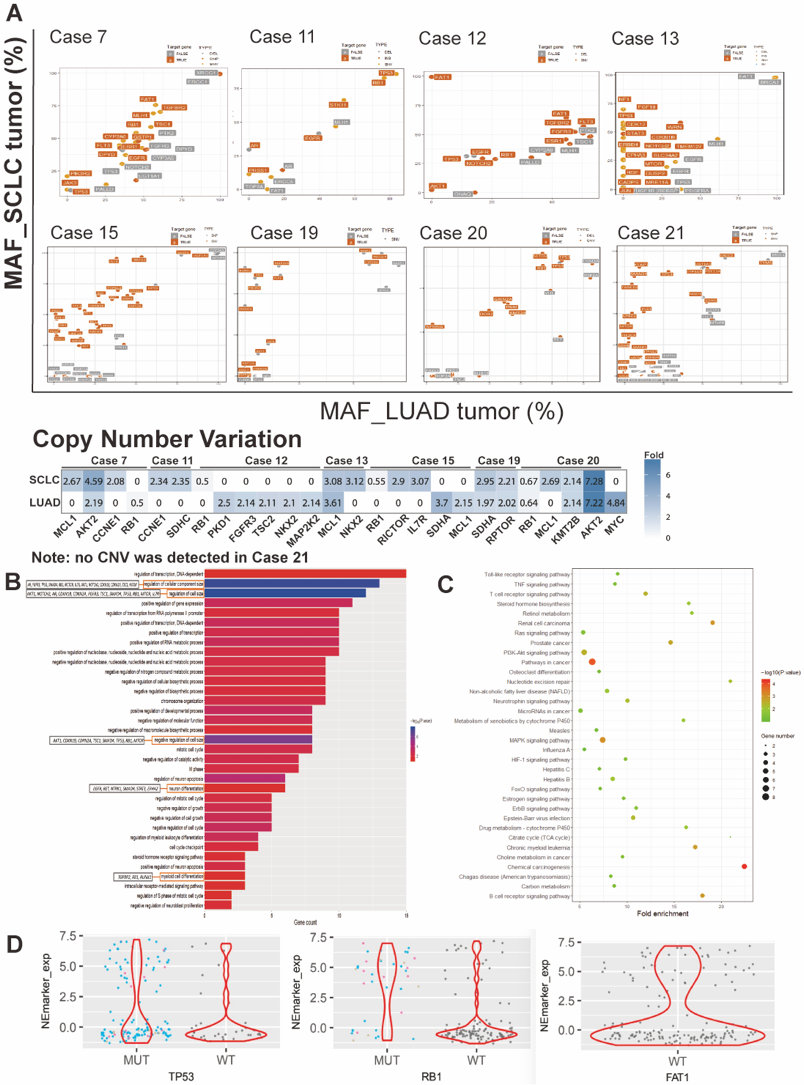
Fig. S1.

A, Altered genes in primary adenocarcinoma and transformed SCLC tumors from individual cases. B-C, pathway enrichment analysis of genes with higher MAF or CNV. D, Violin analysis of associations between TP53, RB1, FAT1 and mRNA expression levels of neuroendocrine related genes. Abbreviations, MUT, mutant, WT, wildtype.


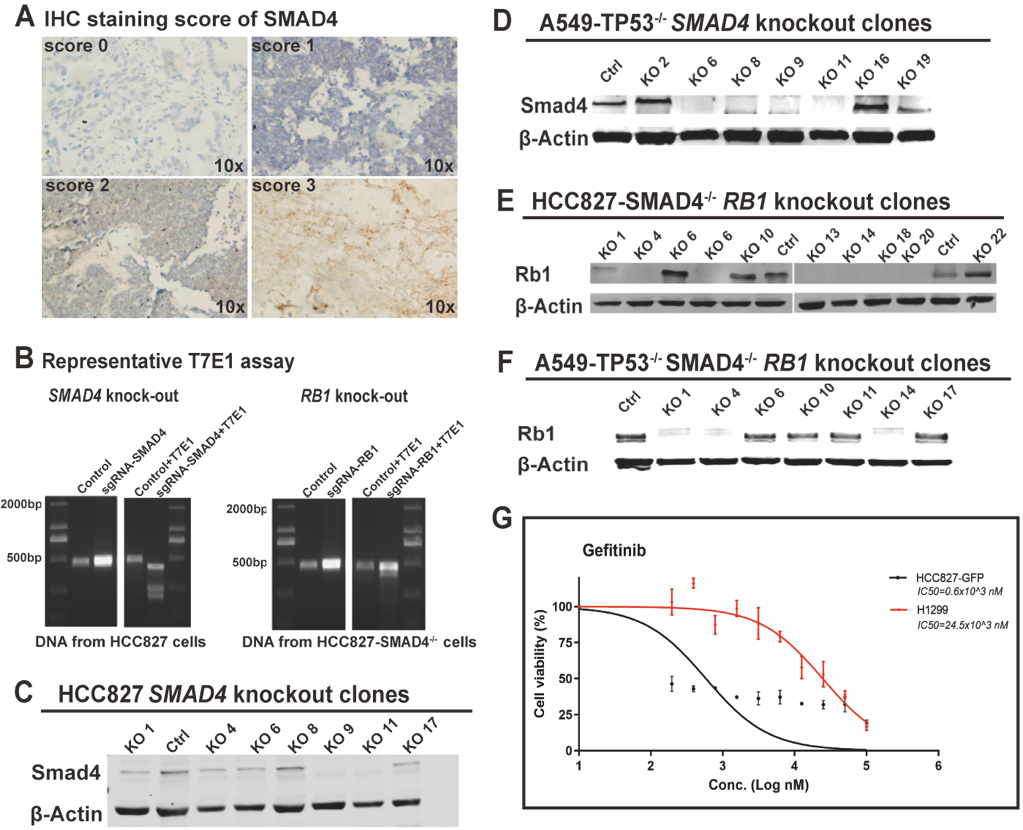
Fig. S2.

A: The intensity scores of cytoplasmic and nuclear Smad4 expression by immunohistochemistry. score 0, negative; score 1, weak; score 2, moderate; score 3, strong. B-F, Establishment of SMAD4 and RB1 knock-out cell models. B: Representative T7E1 mismatch detection assay performed on DNA of HCC827 cells stably transfected with SMAD4 and/or RB1 gRNAs. C-F: Monoclonal screening of SMAD4 and/or RB1 knock-out clones in HCC827 and A549-TP53­­-/- ­­­cells. HCC827-SMAD4 KO 6 (C), A549-TP53­­-/- SMAD4 KO 11 (D), HCC827-SMAD4-/- RB1 KO 13 (E) and A549-TP53­­-/- SMAD4-/- RB1 KO 4 (F) were selected for further study labeled as HCC827-SMAD4-/-, HCC827-SMAD4-/-RB1-/-, A549-TP53-/--SMAD4-/- and A549-TP53-/--SMAD4-/-RB1-/-, respectively. G, Drug sensitivity of Gefitinib, the 1st line EGFR-TKI, in HCC827 (EGFR E746-A750 deletion) and H1299 (EGFR wildtype) cells. The IC50 of gefitinib was 0.6μM and 24.5μM in HCC827 and H1299 cells, respectively.


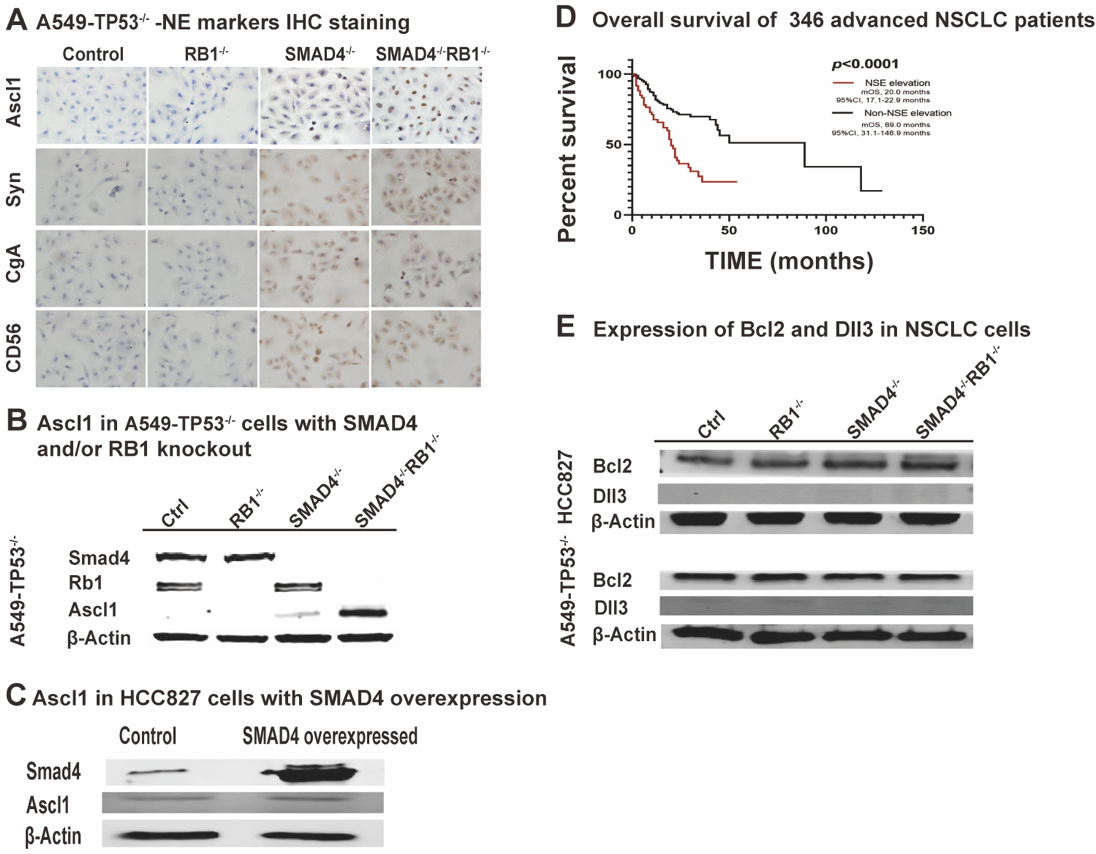
Fig. S3.

A, IHC staining performed on A549-TP53-/- cells. Positive expression of Syn was observed in SMAD4 deleted cells, and no positive staining was found when RB1 was knocked out alone. B, Western blotting performed on proteins extracted from A549-TP53-/- cells. An upregulated expression of Ascl1 was detected in SMAD4 knock-out cell lines but not detected when RB1 was knocked out alone. C, Detection of Ascl1 expression in HCC827 cells with SMAD4 overexpression. No obvious change of Ascl1 expression was detected when SMAD4 was overexpressed in HCC827 cells as compared to GFP control. D, Overall survival curve of 346 advanced NSCLC patients. The prognosis of NSCLC with acquired NSE elevation (n=78) was significantly worse than that of NSCLC without NSE elevation (n=268) (mOS: 20 vs 89 months, P<0.0001). E, Expression of Bcl2 and Dll3 in NSCLC cells. No obvious changes of Bcl2 or Dll3 expression were found when SMAD4 and/or RB1 was inactivated in HCC827 and A549-TP53-/- cells.

Table S1 Sequenced gene list involving 416 genes

| **Gene** | **Gene** | **Gene** | **Gene** | **Gene** | **Gene** | **Gene** |
| --- | --- | --- | --- | --- | --- | --- |
| ABCB1(MDR1) | CDKN1A | ERC1 | HSD3B1 | MTOR | PRSS1 | STRN |
| ABCC2(MRP2) | CDKN1B | ERCC1 | IDH1 | MUTYH | PTCH1 | STT3A |
| ADH1B | CDKN1C | ERCC2 | IDH2 | MYC | PTEN | SUFU |
| AFF1 | CDKN2A | ERCC3 | IGF1R | MYCL | PTK2 | TACC1 |
| AFF4 | CDKN2B | ERCC4 | IGF2 | MYCN | PTPN11 | TACC3 |
| AIP | CDKN2C | ERCC5 | IKBKE | MYD88 | PTPRD | TEK |
| AKT1 | CEBPA | ERG | IKZF1 | NAT1 | QKI | TEKT4 |
| AKT2 | CEP57 | ESR1 | IKZF3 | NBN | RAC1 | TERC |
| AKT3 | CHD4 | ETV1 | IL7R | NCOA4 | RAD50 | TERT |
| ALDH2 | CHEK1 | ETV4 | INPP4B | NF1 | RAD51 | TET2 |
| ALK | CHEK2 | ETV6 | INPP5D | NF2 | RAD51C | TFG |
| AMER1 | CLIP1 | EWSR1 | IRF2 | NFKBIA | RAD51D | TGFBR2 |
| APC | CLTC | EXT1 | JAK1 | NKX2-1 | RAF1 | THADA |
| AR | COL1A1 | EXT2 | JAK2 | NOTCH1 | RARA | TMEM127 |
| ARAF | CREB1 | EZH2 | JAK3 | NOTCH2 | RB1 | TMPRSS2 |
| ARID1A | CREBBP | EZR | JUN | NPM1 | RECQL4 | TNFAIP3 |
| ARID2 | CRKL | FANCA | KDM5A | NQO1 | RET | TNFRSF11A |
| ARID5B | CSF1R | FANCC | KDM6A | NR4A3 | RHOA | TNFRSF14 |
| ASXL1 | CTCF | FANCD2 | KDR(VEGFR2) | NRAS | RICTOR | TNFRSF19 |
| ATF1 | CTLA4 | FANCE | KIF5B | NSD1 | RNF146 | TNFSF11 |
| ATIC | CTNNB1 | FANCF | KIT | NTRK1 | RNF43 | TOP1 |
| ATM | CXCR4 | FANCG | KITLG | PAK3 | ROS1 | TOP2A |
| ATR | CYLD | FANCL | KLC1 | PALB2 | RPTOR | TP53 |
| ATRX | CYP19A1 | FAT1 | KLLN | PALLD | RRM1 | TPM3 |
| AURKA | CYP2A6 | FBX1 | KMT2A | PARK2 | RTEL1 | TPM4 |
| AURKB | CYP2B6*6 | FBXW7 | KMT2B | PARP1 | RUNX1 | TPMT*2 |
| AXIN2 | CYP2C19*2 | FEV | KRAS | PARP2 | SBDS | TPMT*3 |
| AXL | CYP2C9*3 | FGF19 | KTN1 | PAX5 | SDC4 | TPMT*4 |
| BAIAP2L1 | CYP2D6*10 | FGFR1 | LHCGR | PBRM1 | SDHA | TPMT*5 |
| BAK1 | CYP2D6*4 | FGFR2 | LMO1 | PCDH11Y | SDHAF2 | TPMT*6 |
| BAP1 | CYP2D6*5 | FGFR3 | LRIG3 | PDCD1 (PD1) | SDHB | TPMT*7 |
| BARD1 | CYP2D6*6 | FGFR4 | LYN | PDCD1LG2(PD-L2) | SDHC | TPMT*10 |
| BCL2 | CYP2D6*7 | FH | LZTR1 | PDE11A | SDHD | TRIM24 |
| BCL2L11(BIM) | CYP2D6*3 | FLCN | MAP2K1(MEK1) | PDGFRA | SEPT9 | TRIM27 |
| BIRC3 | CYP2D6*12 | FLI1 | MAP2K2(MEK2) | PDGFRB | SERP2 | TRIM33 |
| BLM | CYP2D6*14 | FLT1(VEGFR1) | MAP2K4 | PDK1 | SETBP1 | TSC1 |
| BMPR1A | CYP3A4*4 | FLT3 | MAP3K1 | PGR | SETD2 | TSC2 |
| BRAF | CYP3A5*1 | FLT4 | MAP4K3 | PHOX2B | SF3B1 | TSHR |
| BRCA1 | CYP3A5*3 | GATA1 | MAX | PIK3C3 | SGK1 | TTF1 |
| BRCA2 | DAXX | GATA2 | MCL1 | PIK3CA | SH2D1A | TUBB3 |
| BRD4 | DCTN1 | GATA3 | MDM2 | PIK3R1 | SHOX | TYMS |
| BRIP1 | DDIT3 | GATA4 | MDM4 | PIK3R2 | SLC34A2 | UGT1A1 |
| BTG2 | DDR2 | GATA6 | MED12 | PKD1 | SLC7A8 | VEGFA |
| BTK | DENND1A | GNA11 | MEF2B | PKD2 | SLX4 | VHL |
| BUB1B | DHFR | GNAQ | MEN1 | PKHD1 | SMAD2 | WAS |
| c11orf30 | DICER1 | GNAS | MET | PLAG1 | SMAD3 | WISP3 |
| CBL | DNMT3A | GOLGA5 | MGMT | PLK1 | SMAD4 | WRN |
| CBLB | DPYD | GOPC | MITF | PMS1 | SMAD7 | WT1 |
| CCND1 | DUSP2 | GRIN2A | MLH1 | PMS2 | SMARCA4 | XPA |
| CCNE1 | EGFR | GRM3 | MLH3 | POLD1 | SMARCB1 | XPC |
| CD274(PD-L1) | EML4 | GSTM1 | MLLT1 | POLE | SMO | XRCC1 |
| CD74 | EP300 | GSTP1 | MLLT10 | POLH | SOX2 | YAP1 |
| CDA | EPAS1 | GSTT1 | MLLT3 | POT1 | SPOP | ZNF2 |
| CDC73 | EPCAM | HDAC2 | MLLT4 | POU5F1 | SPRY4 | ZNF217 |
| CDH1 | EPHA2 | HGF | MPL | PPP2R1A | SRC | ZNF444 |
| CDK10 | EPHA3 | HIP1 | MRE11A | PRDM1 | SRY | ZNF703 |
| CDK12 | EPS15 | HLA-A | MSH2 | PRF1 | STAG2 |  |
| CDK4 | ERBB2(HER2) | HNF1A | MSH3 | PRKACA | STAT3 |  |
| CDK6 | ERBB3 | HNF1B | MSH6 | PRKAR1A | STK11 |  |
| CDK8 | ERBB4 | HRAS | MTHFR | PRKCI | STMN1 |  |

Table S2 General sequencing results of 8 SCLC transformation cases

| **sample** | **Symbol** | **Mutation** | **MAF/Copy number gains** | |
| --- | --- | --- | --- | --- |
|  |  |  | **LUAD(before SCHT)** | **SCLC(after SCHT)** |
| Case7 | AKT2 | CNV | 2.190769231 | 4.587692 |
| Case7 | CCNE1 | CNV | 0 | 2.077273 |
| Case7 | CYP2A6 | p.S224P (c.T670C) | 0.3684 | 0.4813 |
| Case7 | CYP3A5 | c.G219-237A | 0.512 | 0.4123 |
| Case7 | DPYD | p.R29C (c.C85T) | 0.5199 | 0.4147 |
| Case7 | DPYD | p.I543V (c.A1627G) | 0.3482 | 0.4229 |
| Case7 | EGFR | p.746_750del (c.2236_2250delGAATTAAGAGAAGCA) | 0.3634 | 0.4091 |
| Case7 | ERCC1 | p.N118N (c.T354C) | 1 | 0.992 |
| Case7 | ESR1 | p.R555H (c.G1664A) | 0.4491 | 0.4688 |
| Case7 | FAT1 | p.L12F (c.C34T) | 0.5789 | 0.7557 |
| Case7 | FGFR3 | p.A500T (c.G1498A) | 0.4886 | 0.4251 |
| Case7 | FLT3 | p.R387P (c.G1160C) | 0.3416 | 0.4448 |
| Case7 | GSTP1 | p.I105V (c.A313G) | 0.4665 | 0.4781 |
| Case7 | JAK3 | p.C54R (c.T160C) | 0 | 0.1061 |
| Case7 | MCL1 | CNV | 0 | 2.67 |
| Case7 | MLH1 | p.V384D (c.T1151A) | 0.5628 | 0.6916 |
| Case7 | NOTCH2 | p.R1332H (c.G3995A) | 0.382 | 0.3717 |
| Case7 | PALLD | p.E54K (c.G160A) | 0.2545 | 0.1461 |
| Case7 | PIK3R2 | p.T487I (c.C1460T) |  | 0.2081 |
| Case7 | PTK2 | p.M783L (c.A2347C) | 0.5409 | 0.4966 |
| Case7 | RB1 | CNV | 0.5 | 0 |
| Case7 | RB1 | p.Y790X (c.C2370A) | 0.4516 | 0.5427 |
| Case7 | TGFBR2 | p.T315M (c.C944T) | 0.6095 | 0.7031 |
| Case7 | TP53 | p.Q331X (c.C991T) | 0.3594 | 0.3041 |
| Case7 | TP53 | c.672+1delG | 0.0227 | 0.1022 |
| Case7 | TSC1 | p.A84T (c.G250A) | 0.5483 | 0.5858 |
| Case7 | UGT1A1 | p.G71R (c.G211A) | 0.4517 | 0.179 |
| Case7 | XRCC1 | p.Q399R (c.A1196G) | 0.9983 | 0.9934 |
| Case11 | AR | p.L57delinsLQQQ (c.170_171insGCAGCAGCA) | 0.1912 | 0.295 |
| Case11 | AR | p.457del (c.1369_1371delGGC) |  | 0.1421 |
| Case11 | CCNE1 | CNV |  | 2.335455 |
| Case11 | EGFR | p.746_750del (c.2236_2250delGAATTAAGAGAAGCA) | 0.3957 | 0.4151 |
| Case11 | ERCC5 | p.E734D (c.G2202C) | 0.1256 | 0.089 |
| Case11 | FAT1 | p.V1373I (c.G4117A) | 0.105 |  |
| Case11 | MLH1 | p.V384D (c.T1151A) | 0.4938 | 0.4674 |
| Case11 | PRSS1 | p.G191R (c.G571A) |  | 0.1107 |
| Case11 | RB1 | p.S816X (c.C2447G) | 0.7672 | 0.827 |
| Case11 | SDHC | CNV |  | 2.348333 |
| Case11 | STK11 | c.C375-8T | 0.5395 | 0.6627 |
| Case11 | TOP2A | p.G1431S (c.G4291A) | 0.0678 | 0.0545 |
| Case11 | TP53 | p.G245D (c.G734A) | 0.8368 | 0.8571 |
| Case12 | AKT1 | p.G162D (c.G485A) |  | 0.0207 |
| Case12 | CYP2A6 | p.S224P (c.T670C) | 0.387596899 | 0.3297 |
| Case12 | EGFR | p.746_750del (c.2236_2250delGAATTAAGAGAAGCA) | 0.142857143 | 0.3128 |
| Case12 | EGFR | p.746_750del (c.2236_2250delGAATTAAGAGAAGCA) | |  |
| Case12 | ESR1 | p.R555H (c.G1664A) | 0.447154472 | 0.455 |
| Case12 | FAT1 | p.L12F (c.C34T) | 0.449275362 | 0.6404 |
| Case12 | FAT1 | p.Q2933P (c.A8798C) |  | 0.9914 |
| Case12 | FGFR3 | p.A500T (c.G1498A) | 0.4778157 | 0.5201 |
| Case12 | FGFR3 | CNV | 2.138888889 |  |
| Case12 | FLT3 | p.R387P (c.G1160C) | 0.50617284 | 0.5678 |
| Case12 | GNAQ | p.T96S (c.A286T) | 0.144927536 |  |
| Case12 | MAP2K2 | CNV | 2.142727273 |  |
| Case12 | MLH1 | p.V384D (c.T1151A) | 0.461538462 | 0.4167 |
| Case12 | NKX2-1 | CNV | 2.096666667 |  |
| Case12 | NOTCH2 | p.R1332H (c.G3995A) | 0.169811321 | 0.2921 |
| Case12 | PALLD | p.E54K (c.G160A) | 0.333333333 | 0.3023 |
| Case12 | PKD1 | CNV | 2.497391304 |  |
| Case12 | PTK2 | p.M783L (c.A2347C) | 0.53030303 | 0.4811 |
| Case12 | RB1 | p.Y790X (c.C2370A) | 0.212389381 | 0.287 |
| Case12 | RB1 | CNV |  | 0.5 |
| Case12 | TGFBR2 | p.T315M (c.C944T) | 0.504132231 | 0.5075 |
| Case12 | TP53 | c.672+1delG | 0.117241379 | 0.3125 |
| Case12 | TSC1 | p.A84T (c.G250A) | 0.527027027 | 0.4766 |
| Case12 | TSC2 | CNV | 2.105365854 |  |
| Case13 | BRCA1 | p.S1613G (c.A4837G) | 0.9844 | 0.9744 |
| Case13 | CADPS | SV |  | 0.02 |
| Case13 | CDK12 | p.V896fs (c.2687delT) |  | 0.5333 |
| Case13 | CDKN1B | p.P69fs (c.205_206insTA) |  | 0.4844 |
| Case13 | CREBBP | p.D1273Y (c.G3817T) | 0.1765 |  |
| Case13 | CTNNB1 | p.S33C (c.C98G) | 0.0497 |  |
| Case13 | DUSP2 | p.R160S (c.C478A) |  | 0.1908 |
| Case13 | EGFR | p.S768I (c.G2303T) | 0.2837 | 0.1937 |
| Case13 | EGFR | p.G719C (c.G2155T) | 0.3387 | 0.2387 |
| Case13 | EPHA3 | p.Q607K (c.C1819A) |  | 0.3375 |
| Case13 | ERBB4 | p.P1196H (c.C3587A) |  | 0.3545 |
| Case13 | FAT1 | p.Q2933P (c.A8798C) | 0.9922 | 0.9753 |
| Case13 | FGF19 | p.R63S (c.C187A) |  | 0.581 |
| Case13 | HGF | p.R328C (c.C982T) |  | 0.1882 |
| Case13 | IGF1R | p.C1059Y (c.G3176A) | 0.0723 |  |
| Case13 | JUN | p.H52Q (c.C156G) |  | 0.0148 |
| Case13 | MCL1 | CNV | 3.606666667 | 3.083333 |
| Case13 | MLH1 | p.K722E (c.A2164G) | 0.6136 | 0.4382 |
| Case13 | MRE11A | p.L240P (c.T719C) |  | 0.117 |
| Case13 | MTOR | p.D138Y (c.G412T) |  | 0.254 |
| Case13 | NF1 | p.K205X (c.A613T) |  | 0.6966 |
| Case13 | NKX2-1 | CNV |  | 3.12 |
| Case13 | NOTCH2 | p.C347S (c.G1040C) |  | 0.4483 |
| Case13 | PDGFRA | p.G255C (c.G763T) | 0.375 |  |
| Case13 | SLC34A2 | p.L690S (c.T2069C) |  | 0.2604 |
| Case13 | STAT3 | p.I597V (c.A1789G) |  | 0.5281 |
| Case13 | TMEM127 | p.Y74C (c.A221G) |  | 0.3333 |
| Case13 | TP53 | p.R158L (c.G473T) | 0.3333 | 0.5606 |
| Case13 | WRN | p.P1365fs (c.4093_4094insA) | 0.3714 | 0.5833 |
| Case15 | ABCB1 | p.N21S (c.A62G) | 0.2692 | 0.3867 |
| Case15 | ALK | p.Q459K (c.C1375A) | 0.2709 | 0.6533 |
| Case15 | BRCA2 | p.K2729N (c.G8187T) | 0.534 | 0.9695 |
| Case15 | CDA | p.K27Q (c.A79C) | 0.3808 | 0.3065 |
| Case15 | CDKN1B | p.G182C (c.G544T) | 0.2746 | 0.5953 |
| Case15 | CYLD | p.L460F (c.G1380T) | 0.0128 |  |
| Case15 | DAXX | p.L500P (c.T1499C) | 0.2189 |  |
| Case15 | DPYD | p.I543V (c.A1627G) | 0.6189 | 0.7406 |
| Case15 | EPHA2 | c.T979+2A | 0.484 | 0.6705 |
| Case15 | FLT4 | p.H379Q (c.C1137A) | 0.3816 | 0.9569 |
| Case15 | GNAS | p.P47T (c.C139A) | 0.2835 |  |
| Case15 | GNAS | p.P109H (c.C326A) | 0.4471 | 0.6562 |
| Case15 | GSTP1 | p.I105V (c.A313G) | 0.7752 | 0.9819 |
| Case15 | HNF1A | p.Q561L (c.A1682T) | 0.1478 | 0.3731 |
| Case15 | IL7R | CNV |  | 3.07 |
| Case15 | JAK1 | p.M641L (c.A1921T) | 0.354 | 0.6212 |
| Case15 | KDM5A | p.A1556L (c.GC4666TT) | 0.015 |  |
| Case15 | KMT2A | p.E887X (c.G2659T) | 0.073 |  |
| Case15 | KMT2B | p.P2140L (c.C6419T) | 0.5089 | 0.5947 |
| Case15 | LHCGR | p.A430D (c.C1289A) | 0.1623 | 0.3066 |
| Case15 | MCL1 | CNV | 2.146666667 |  |
| Case15 | MTHFR | p.A222V (c.C665T) | 0.9923 | 0.989 |
| Case15 | MTOR | p.S1276C (c.C3827G) | 0.0182 |  |
| Case15 | NF1 | c.G4431-1T | 0.1872 | 0.5322 |
| Case15 | NF1 | p.Q835X (c.C2503T) | 0.2608 | 0.422 |
| Case15 | PKD1 | p.A2345S (c.G7033T) | 0 | 0.5035 |
| Case15 | PKHD1 | p.P383L (c.C1148T) | 0.0632 |  |
| Case15 | POLE | p.N1816K (c.C5448A) | 0 | 0.3516 |
| Case15 | PRKCI | p.Q576R (c.A1727G) | 0.2085 |  |
| Case15 | PTCH1 | p.G1072D (c.G3215A) | 0.0099 |  |
| Case15 | PTPN13 | p.R895W (c.A2683T) | 0.3891 | 0.6951 |
| Case15 | RAF1 | p.S257L (c.C770T) | 0 | 0.4627 |
| Case15 | RB1 | p.Q850X (c.C2548T) | 0 | 0.3771 |
| Case15 | RB1 | CNV |  | 0.55 |
| Case15 | RECQL4 | p.R98H (c.G293A) | 0.0131 |  |
| Case15 | RET | p.A301E (c.C902A) | 0.1525 | 0.2479 |
| Case15 | RICTOR | CNV |  | 2.9 |
| Case15 | SDHA | CNV | 3.7 |  |
| Case15 | SGK1 | p.L382P (c.T1145C) | 0 | 0.3263 |
| Case15 | STK11 | p.F354L (c.C1062G) | 0.426 | 0.2583 |
| Case15 | TP53 | p.E271X (c.G811T) | 0.1574 | 0.4039 |
| Case15 | TP53 | p.R158L (c.G473T) | 0.2784 | 0.5489 |
| Case15 | UGT1A1 | p.G71R (c.G211A) | 0.9795 | 0.9957 |
| Case19 | AKT2 | p.H89L (c.A266T) | 0 | 0.1073 |
| Case19 | ASXL1 | p.G659S (c.G1975A) |  | 0.0125 |
| Case19 | BTG2 | p.R66C (c.C196T) | 0.013 |  |
| Case19 | CDKN2A | p.R131C (c.C391T) |  | 0.0104 |
| Case19 | CYP2D6 | p.G169R (c.G505A) | 0.4583 | 0.5421 |
| Case19 | FAT1 | p.D3360fs (c.10078delG) | 0.0852 | 0.1551 |
| Case19 | FLT4 | p.V1097M (c.G3289A) | 0.1304 | 0.4909 |
| Case19 | KMT2A | p.S518F (c.C1553T) |  | 0.0412 |
| Case19 | KMT2B | p.R538H (c.G1613A) |  | 0.0122 |
| Case19 | MAP2K4 | p.K350N (c.G1050T) | 0.1244 | 0.5347 |
| Case19 | MSH2 | p.Q629R (c.A1886G) | 0.4378 | 0.5979 |
| Case19 | NF1 | p.Q1972X (c.C5914T) | 0.0364 |  |
| Case19 | PIK3R2 | p.Q289K (c.C865A) | 0 | 0.4441 |
| Case19 | RAD51 | p.N34T (c.A101C) | 0.5669 | 0.5369 |
| Case19 | RB1 | p.K294X (c.A880T) | 0 | 0.4762 |
| Case19 | RECQL4 | p.T449P (c.A1345C) | 0.5042 | 0.5972 |
| Case19 | RPTOR | CNV | 2.016395154 | 2.210463 |
| Case19 | RUNX1 | p.L445P (c.T1334C) | 0.5149 | 0.5283 |
| Case19 | SDHA | CNV | 1.969262724 | 2.949762 |
| Case19 | SETD2 | p.Q1466E (c.C4396G) | 0.5209 | 0.4327 |
| Case19 | SMAD4 | p.Q289X (c.C865T) |  | 0.3421 |
| Case19 | TUBB6 | p.V353M (c.G1057A) | 0.0125 |  |
| Case20 | AKT2 | CNV | 7.221484196 | 7.284104 |
| Case20 | BUB1B | p.Q827E (c.C2479G) | 0.1678 |  |
| Case20 | DDR2 | p.R680H (c.G2039A) | 0.199 | 0.3004 |
| Case20 | GRIN2A | p.P1430S (c.C4288T) | 0.2476 | 0.3364 |
| Case20 | KMT2B | p.G1999W (c.G5995T) | 0.2735 | 0.2798 |
| Case20 | KMT2B | CNV | 2.143060225 | 2.138407 |
| Case20 | MCL1 | CNV |  | 2.694444 |
| Case20 | MYC | CNV | 4.837578838 |  |
| Case20 | NFKBIA | p.G161A (c.G482C) | 0 | 0.2371 |
| Case20 | PALB2 | p.R170X (c.A508T) | 0.2592 | 0.3288 |
| Case20 | PKD1 | p.H267Q (c.C801G) | 0.0816 | 0 |
| Case20 | PRKCI | p.R166H (c.G497A) | 0.0165 |  |
| Case20 | RB1 | CNV | 0.64 | 0.67 |
| Case20 | RET | p.R114H (c.G341A) | 0.4263 | 0.1799 |
| Case20 | SETD2 | p.E639Q (c.G1915C) | 0.3693 | 0.5602 |
| Case20 | THADA | p.Q209P (c.A626C) | 0.5281 | 0.5109 |
| Case20 | TOP2A | p.I43T (c.T128C) | 0.0263 |  |
| Case20 | TP53 | p.A86fs (c.256delG) | 0.4237 | 0.5159 |
| Case20 | TP53 | p.P85L (c.C254T) | 0.4308 | 0.5241 |
| Case20 | TSC2 | c.G2837+12C | 0.0816 |  |
| Case20 | VHL | p.R167Q (c.G500A) | 0.3952 | 0.3908 |
| Case21 | ABCB1 | p.E1201K (c.G3601A) | 0.0565 |  |
| Case21 | AMER1 | p.A449G (c.C1346G) |  | 0.0355 |
| Case21 | ARID2 | p.Q1128X (c.C3382T) | 0.218 | 0 |
| Case21 | BRCA2 | p.S3094L (c.C9281T) | 0.0263 |  |
| Case21 | BUB1B | p.D377H (c.G1129C) | 0.0561 |  |
| Case21 | CBLB | p.C224Y (c.G671A) | 0.0233 |  |
| Case21 | CREBBP | p.Q771X (c.C2311T) | 0.0286 |  |
| Case21 | CYP3A5 | c.G219-237A | 0.4955 | 0.86 |
| Case21 | EPHA2 | p.D799N (c.G2395A) |  | 0.0495 |
| Case21 | EPHA3 | p.T166N (c.C497A) | 0.1834 | 0 |
| Case21 | ERCC1 | p.N118N (c.T354C) | 1 | 0.996 |
| Case21 | ERCC2 | p.K751Q (c.A2251C) | 0.6462 | 0.9448 |
| Case21 | FANCD2 | p.G333E (c.G998A) | 0 | 0.7366 |
| Case21 | GNAS | p.G716C (c.G2146T) |  | 0.0174 |
| Case21 | GRIN2A | p.T1262S (c.C3785G) |  | 0.0155 |
| Case21 | GSTP1 | p.I105V (c.A313G) | 0.5491 | 0.5021 |
| Case21 | KEAP1 | p.H311R (c.A932G) | 0 | 0.8703 |
| Case21 | KEAP1 | p.H311R (c.A932G) |  |  |
| Case21 | KMT2A | p.K3230N (c.G9690C) | 0.0268 |  |
| Case21 | KMT2A | p.Q3433X (c.C10297T) | 0.0444 |  |
| Case21 | LYN | p.R156T (c.G467C) | 0.0241 |  |
| Case21 | MED12 | p.E1719K (c.G5155A) | 0.0545 |  |
| Case21 | MTHFR | p.A222V (c.C665T) | 0.5705 | 0.4513 |
| Case21 | MTOR | p.I458M (c.C1374G) | 0 | 0.4222 |
| Case21 | NQO1 | p.P187S (c.C559T) | 0.5035 | 0.6309 |
| Case21 | NTRK1 | p.D674Y (c.G2020T) | 0 | 0.5049 |
| Case21 | PDE11A | p.S604X (c.C1811G) | 0.5323 | 0.863 |
| Case21 | PIK3CA | p.E545K (c.G1633A) | 0 | 0.3043 |
| Case21 | PLK1 | p.E206X (c.G616T) | 0.1229 | 0.5134 |
| Case21 | RB1 | p.G100X (c.G298T) |  | 0.0292 |
| Case21 | RET | p.L362I (c.C1084A) | 0.3657 | 0 |
| Case21 | ROS1 | p.T1332P (c.A3994C) |  | 0.0247 |
| Case21 | SDHB | p.C192Y (c.G575A) | 0 | 0.2685 |
| Case21 | SDHD | p.D118E (c.T354G) | 0.5263 | 0.5911 |
| Case21 | SETBP1 | p.M1319I (c.G3957A) | 0.0496 |  |
| Case21 | SMAD4 | p.W524C (c.G1572T) | 0 | 0.7969 |
| Case21 | SOS1 | p.S548R (c.T1644A) |  | 0.0103 |
| Case21 | TET2 | p.E1144K (c.G3430A) | 0.1116 |  |
| Case21 | TP53 | p.G154V (c.G461T) | 0.2815 | 0.8382 |
| Case21 | TPMT | p.R215C (c.C643T) | 0.0262 |  |
| Case21 | TSC1 | p.Q654E (c.C1960G) | 0.559 | 0.455 |
| Case21 | TYMS | c.X447_X452delTTAAAG | 0.9221 | 0.9319 |
| Case21 | UGT1A1 | p.G71R (c.G211A) | 0.5089 | 0.875 |

**Table S3 Classic inherited gene alterations in SCLC transformation from EGFR-wildtype NSCLC**

| **Case ID** | **Symbol** | **Genetic alterations** | |
| --- | --- | --- | --- |
|  |  | Before transformation  (NSCLC component) | After transformation  (SCLC component) |
| Case 15 | *ALK* | p.Q459K (c.C1375A) | p.Q459K (c.C1375A) |
|  | *TP53* | p.R158L (c.G473T)  p.E271X (c.G811T) | p.R158L (c.G473T)  p.E271X (c.G811T) |
|  | *RB1* | p.Q850X (c.C2548T) | p.Q850X (c.C2548T)  copy number variation |
| Case 19 | *RB1* | p.K294X (c.A880T) | p.K294X (c.A880T) |
| Case 20 | *TP53* | p.P85L (c.C254T)  p.A86fs (c.256delG) | p.P85L (c.C254T)  p.A86fs (c.256delG) |
|  | *RB1* | copy number variation | copy number variation |
| Case 21 | *TP53* | p.G154V (c.G461T) | p.G154V (c.G461T) |
